# Microbial Metabolite Inspired *β*‐Peptide Polymers Displaying Potent and Selective Antifungal Activity

**DOI:** 10.1002/advs.202104871

**Published:** 2022-03-20

**Authors:** Donghui Zhang, Chao Shi, Zihao Cong, Qi Chen, Yufang Bi, Junyu Zhang, Kaiqian Ma, Shiqi Liu, Jiawei Gu, Minzhang Chen, Ziyi Lu, Haodong Zhang, Jiayang Xie, Ximian Xiao, Longqiang Liu, Weinan Jiang, Ning Shao, Sheng Chen, Min Zhou, Xiaoyan Shao, Yidong Dai, Maoquan Li, Lixin Zhang, Runhui Liu

**Affiliations:** ^1^ State Key Laboratory of Bioreactor Engineering East China University of Science and Technology Shanghai 200237 China; ^2^ Key Laboratory for Ultrafine Materials of Ministry of Education Frontiers Science Center for Materiobiology and Dynamic Chemistry Research Center for Biomedical Materials of Ministry of Education School of Materials Science and Engineering East China University of Science and Technology Shanghai 200237 China; ^3^ Shanghai Ruijin Rehabilitation Hospital Shanghai 200023 China; ^4^ Department of Interventional and Vascular Surgery Shanghai Clinical Research Center for Interventional Medicine Shanghai Tenth People's Hospital Tongji University School of Medicine Shanghai 200072 China

**Keywords:** antifungal agents, fungal keratitis, host defense peptide mimicking, poly(DL‐diaminopropionic acid), *β*‐peptide polymers

## Abstract

Potent and selective antifungal agents are urgently needed due to the quick increase of serious invasive fungal infections and the limited antifungal drugs available. Microbial metabolites have been a rich source of antimicrobial agents and have inspired the authors to design and obtain potent and selective antifungal agents, poly(DL‐diaminopropionic acid) (PDAP) from the ring‐opening polymerization of *β*‐amino acid *N*‐thiocarboxyanhydrides, by mimicking *ε*‐poly‐lysine. PDAP kills fungal cells by penetrating the fungal cytoplasm, generating reactive oxygen, and inducing fungal apoptosis. The optimal PDAP displays potent antifungal activity with minimum inhibitory concentration as low as 0.4 µg mL^−1^ against *Candida albicans*, negligible hemolysis and cytotoxicity, and no susceptibility to antifungal resistance. In addition, PDAP effectively inhibits the formation of fungal biofilms and eradicates the mature biofilms. In vivo studies show that PDAP is safe and effective in treating fungal keratitis, which suggests PDAPs as promising new antifungal agents.

## Graphical abstract

Inspired by *ε*‐poly‐lysine produced from the fermentation of *Streptomyces albulus*, the *β*‐peptide polymer poly(DL‐diaminopropionic acid) (PDAP) bearing much higher charge density than *ε*‐poly‐lysine, is designed. PDAP is synthesized via the moisture tolerant polymerization of *β*‐amino acid *N*‐thiocarboxyanhydrides. Compared to *ε*‐poly‐lysine that has weak antifungal activity, PDAP displays potent and selective antifungal activity and strong therapeutical potential.

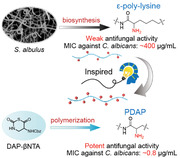

## 1 Introduction

Invasive fungal infections caused 1.5 million deaths worldwide each year, of which 30–40% are *Candida* infections and 20–30% are through *Cryptococcosis*.^[^ ^1^ ^]^ These infections are very common in immunosuppressed populations such as patients who suffer from anti‐cancer chemotherapy, organ transplants, HIV infections, or long‐term use of hormones.^[^ ^2^ ^]^ Both fungi and mammalian cells are eukaryotic organisms and it is extremely difficult to find an antifungal drug with high selectivity or low toxicity. Currently, only a few classes of antifungal drugs are available but commonly with high toxicity and side effects.^[^ ^3^ ^]^ Therefore, the quick emergence of drug‐resistant pathogenic fungi has been a serious threat to human health.^[^ ^4^ ^]^ It is in urgent need to develop potent and nontoxic antifungal agents with promising therapeutic potential.

Microbial metabolites have been a rich source to explore antimicrobial agents.^[^ ^5^ ^]^ *ε*‐poly‐lysine (*ε*‐PL) was discovered from the fermentation of *Streptomyces albulus* in 1977,^[^ ^6^ ^]^ and has been widely used as an FDA‐approved food preservative because of its antimicrobial property.^[^ ^7^ ^]^ Although *ε*‐PL is active against multiple types of bacteria, its activity against fungi is very mild and far from the requirement of an antifungal agent for therapeutic application. Cationic peptides are known to exert antimicrobial activity via initial Coulombic interaction with the negatively charged microbial cell membrane.^[^ ^8^ ^]^ The outer membrane of fungi has lower density of negatively charged lipid than does the outer membrane of bacteria, which causes cationic peptides to have weaker interactions with fungal membranes, and therefore, weaker activities against fungi than bacteria.^[^ ^9^ ^]^ This explains finding of promising cationic antibacterial peptides and peptide mimics,^[^ ^10^ ^]^ but very few promising cationic antifungal agents.^[^ ^11^ ^]^ These inspired us to hypothesize that modifying the structure of *ε*‐PL by increasing the charge density (the charge density per molecular weight of the repeating unit) along the polymer could enhance the interaction between cationic peptides and fungal membrane to enable the finding of potent antifungal agents.

**Figure 1:**
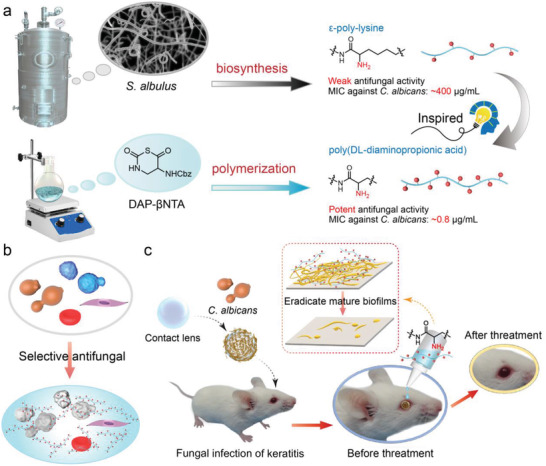
Design and antifungal activities of PDAP. a) *ε*‐poly‐lysine inspired PDAP synthesized from DAP‐*β*‐NTA ring‐opening polymerization. b,c) Schematic diagram of the PDAP that has b) selective antifungal activity, c) eliminates mature biofilms and cures fungal keratitis.

By reducing the carbon number on the backbone of *ε*‐PL from six to three, we design *β*‐peptide polymer poly(DL‐diaminopropionic acid) (PDAP) that has substantially increased charge density than *ε*‐PL (**Figure** **1**a). PDAP can be easily synthesized via the moisture tolerant polymerization of *β*‐amino acid *N*‐thiocarboxyanhydrides (*β*‐NTA) in our recent report.^[^ ^12^ ^]^ Our study indicates that optimal PDAP (a 20 mer PDAP, PDAP_20_) is a promising antifungal agent by demonstrating potent antifungal activity against clinically isolated *Candida albicans* (*C. albicans*) and *Cryptococcus neoformans* (*C. neoformans*), negligible hemolysis and cytotoxicity, insusceptible to antifungal resistance, and promising therapeutic potential in vivo (Figure 1b,c).

## 2 PDAPs Display Potent and Selective Antifungal Activity

**Figure 2:**
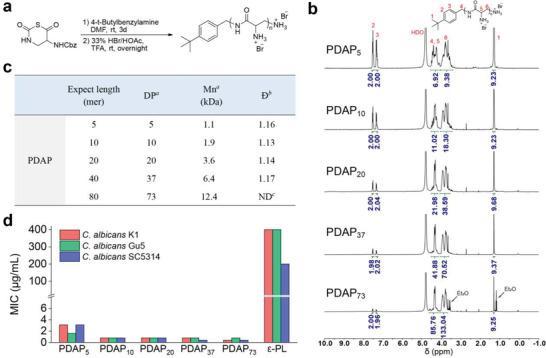
Synthesis, characterization, and activity of PDAP. a) Synthetic scheme of PDAP. b) ^1^H NMR spectra of PDAPs at five different lengths in D_2_O, 400 MHz. c) Summary of PDAP characterization. *^a^* Average degree of polymerization (DP) and Mn were characterized by ^1^H NMR. *^b^* Dispersity, characterized by GPC. Cbz‐protected PDAP at variable length using DMF as the mobile phase at a flow rate of 1 mL min^−1^. *^c^* Not determined, the polymer cannot dissolve thoroughly to conduct accurate GPC characterization. d) Minimum inhibitory concentration (MIC) of PDAP and *ε*‐PL against three strains of *C. albican*s. The molecular weight of purchased *ε*‐PL is *M*_w_ = 3.5–4.6 kDa, which is similar to that of PDAP_20_ (*M*_w_ = 4.1 kDa, *Đ* = 1.14).

The *β*‐peptide polymer PDAPs were synthesized using the water insensitive *β*‐NTA ring‐opening polymerization method,^[^ ^12^ ^]^ followed by deprotection under acidic condition to give the final PDAPs (**Figure** **2**a). To explore the antifungal activity and toxicity of PDAPs, we synthesized PDAPs with five different lengths (DP = 5, 10, 20, 37, 73) and with a narrow dispersity (*Ð*) of 1.13–1.17, as characterized by proton nuclear magnetic resonance (NMR) and gel permeation chromatography (GPC) (Figure 2b,c). In the initial antifungal activity test, we evaluated PDAPs with variable chain length against *C. albicans*, the most common human fungal pathogen, using the minimum inhibitory concentration (MIC). All five PDAPs displayed potent antifungal activities against three strains of *C. albicans* with MIC in a range of 0.4–3.1 µg mL^−1^; in sharp contrast, *ε*‐PL (*M*_w_ = 3.5–4.6 kDa) showed only mild active against *C. albicans*, with MIC at 200–400 µg mL^−1^ (Figure 2d). This result was consistent with the generally low antifungal activity of *ε*‐PL and supported our rational design of PDAPs as potent antifungal agents, which encouraged us to further explore PDAPs as new antifungal agents.

**Figure 3:**
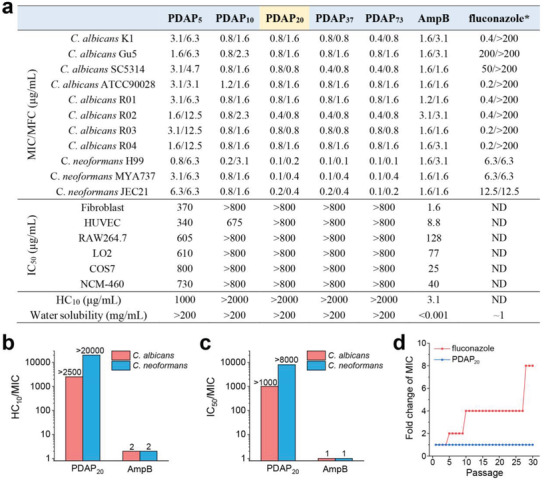
PDAP show potent antifungal activity, low toxicity, and no susceptibility to antifungal resistance. a) Biological activity of PDAPs and antifungal agents, including MIC, minimum fungicidal concentration (MFC), 10% hemolysis (HC_10_), 50% inhibitory concentration for cell viability (IC_50_), and water solubility. * MIC_50_ was used for fluconazole because it cannot inhibit 100% growth of *C. albicans*. b,c) Selectivity index was calculated by the ratio of b) HC_10_ to MIC (*C. albicans* K1 strain) and c) by the ratio of IC_50_ to MIC (*C. albicans* K1 strain). d) Drug resistant assay of PDAP_20_ and fluconazole against *C. albicans* (R02 strain) using continuous treatment of *C. albicans* cells with 0.5 × MIC of PDAP_20_ and 0.5 × MIC_50_ of fluconazole.

We tested PDAPs for their activities against eight strains of *C. albicans* and three strains of *C. neoformans*, using antifungal drugs amphotericin B (AmpB) and fluconazole for comparison. Among these seven fungi strains (K1, Gu5, SC5314, R01, R02, R03, R04) are clinically isolated pathogens. We found that all PDAPs showed potent antifungal activities against these fungi, and the activities increased with the increase of polymer length till reaching a plateau at 20 mer (**Figure** **3**a). PDAP_20_ (DP = 20) displayed MIC at 0.1–0.8 µg mL^−1^ against all eight strains of *C. albicans* and *C. neoformans*, even superior to AmpB with MIC at 1.2–3.1 µg mL^−1^. MIC_50_ was used for fluconazole because it cannot inhibit 100% growth of *C. albicans*, therefore, a 50% inhibition of fungal growth has been widely used for fluconazole.^[^ ^13^ ^]^ Notably, all strains of *C. albicans* still grew even at a high concentration of fluconazole (MIC > 200 µg mL^−1^). Moreover, PDAPs were not only fungistatic but also fungicidal, with the minimum fungicidal concentration (MFC) at 0.2–1.6 µg mL^−1^ against all eight strains of *C. albicans* and *C. neoformans*.

The hemolysis of PDAPs upon human red blood cells was evaluated using the minimum concentration to cause 10% hemolysis (HC_10_). PDAP_5_ showed HC_10_ at 1000 µg mL^−1^ and other PDAPs showed negligible hemolysis up to 2000 µg mL^−1^, with HC_10_ greater than 2000 µg mL^−1^ (Figure 3a; Figure S1a, Supporting Information); whereas, AmpB is hemolytic with HC_10_ at 3.1 µg mL^−1^ (Figure 3a; Figure S1b, Supporting Information). We also evaluated the cytotoxicity of PDAPs upon various mammalian cells including NIH 3T3 fibroblast cells, endothelial cells (HUVEC), macrophage (RAW264.7), liver cells (LO2), kidney cells (COS7) and enterocyte (NCM‐460), using the concentration to cause 50% cell death (IC_50_). PDAP_5_ showed IC_50_ at 340–800 µg mL^−1^ and other PDAPs showed low cytotoxicity up to 800 µg mL^−1^, except PDAP_10_ against HUVEC (IC_50_ = 675 µg mL^−1^) (Figure 3a; Figure S2, Supporting Information); whereas, AmpB is cytotoxic, with IC_50_ at 1.6 µg mL^−1^ for fibroblast cells and 8.8–128 µg mL^−1^ for others (Figure 3a; Figure S2, Supporting Information). All PDAPs are well soluble in water at over 200 mg mL^−1^ compared to the poorly soluble AmpB at <0.001 mg mL^−1^ and moderate soluble fluconazole at ≈1 mg mL^−1^. Among all PDAPs with variable length (DP = 5, 10, 20, 37, 73), PDAP_20_ (DP = 20) is the minimum length to show potent antifungal activities and low toxicity, therefore, was further explored for its biological performance and therapeutic potential. The antifungal selectivity indexes, HC_10_/MIC and IC_50_/MIC, were calculated for PDAP_20_ as higher than 2500 and 1000, respectively, in sharp contrast to that of AmpB at 2 and 1, respectively (Figure 3b,c). These results indicated that PDAP_20_ has superior fungi versus mammalian cell selectivity, superior to AmpB.

In the complex physiological environment, the positively charged salt could compete with antimicrobial peptides by binding to the membrane to antagonize antimicrobial activity.^[^ ^14^ ^]^ To examine this effect in our study, we measured the MIC values of PDAP_20_ under the physiological salt concentrations containing 150 × 10^−3^ m Na^+^ and 5.4 × 10^−3^ m K^+^. The results showed that PDAP_20_ retained potent antifungal activity (MIC = 1.6 µg mL^−1^) under the physiological salt concentrations, only slightly attenuated compared to standard MIC test in RPMI 1640 (MIC = 0.8 µg mL^−1^) (Table S1, Supporting Information). A further increase of Na^+^ concentration from 150× 10^−3^ to 300 × 10^−3^ m resulted in gradually attenuated but still potent antifungal activity of PDAP_20_ (MIC = 1.6–6.3 µg mL^−1^); whereas, the MIC value of *ε*‐PL changed from 400 to >1600 µg mL^−1^ with the increase of Na^+^ concentration to 200 × 10^−3^ m (Table S1, Supporting Information). Increasing of K^+^ concentration up to 20 × 10^−3^ m didn't affect the antifungal activity of either PDAP_20_ or *ε*‐PL. These results showed that PDAP_20_ has excellent tolerance to physiological and environmental monovalent free ions. We also performed the stability test on PDAP_20_ at conditions of salt ions, acid and base using ^1^H NMR. After PDAP_20_ was treated with saturated NaCl, 0.1 n HCl (for pH = 1 condition) and 0.1 m NaOH (for pH = 14 condition), no significant decomposition was found by using ^1^H NMR characterization (Figure S3, Supporting Information).

Resistance to antifungal drugs has been characterized in most fungal species that infect humans, which are emerging as an important clinical problem, especially azole‐resistance.^[^ ^15^ ^]^ We found that a clinically isolated *C. albicans* R02 strain started to adopt drug resistance after five passages of fluconazole treatment, and the MIC_50_ value of fluconazole increased eightfolds after 30 passages, from 0.2 to 1.6 µg mL^−1^. We examined the possible antifungal resistance of PDAP_20_ and found that fungi didn't acquire resistance even after fungal cells were treated with PDAP_20_ continuously over 30 passages (Figure 3d).

To figure out whether the increased charge density affect the selectivity over other microorganisms, we tested the antimicrobial activity of PDAP_20_ against representative Gram‐positive bacteria (*S. aureus, S. epidermidis, B. subtilis*) and Gram‐negative bacteria (*E. coli, A. baumannii, K. pneumoniae*), comparing with *ε*‐PL. The results showed that PDAP_20_ has low antibacterial activities to all above bacteria, with MIC values greater than 50 µg mL^−1^ (Table S2, Supporting Information). In contrast, *ε*‐PL showed higher antibacterial activities, with MIC values in the range of 1.6–6.3 µg mL^−1^. These results demonstrated that PDAP_20_ has high antifungal selectivity, while *ε*‐PL has high antibacterial selectivity, indicating that polymers with high charge density may be more suitable for antifungal agents.

## 3 Antifungal Mechanism of PDAP

**Figure 4:**
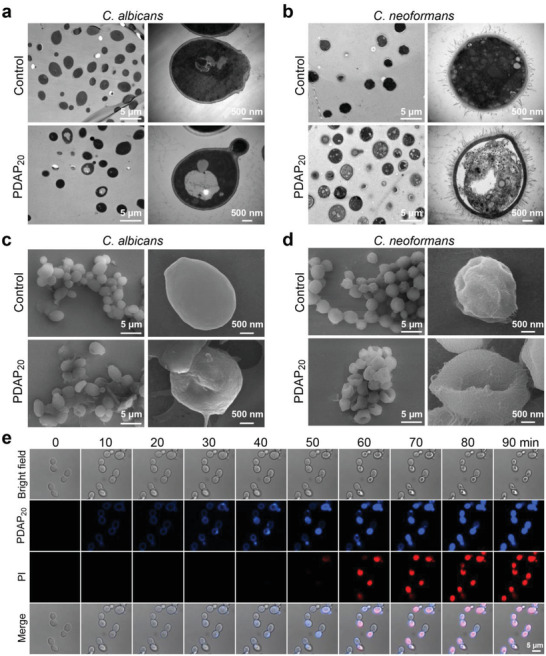
Characterization of fungal cells before and after PDAP_20_ treatment. a,b) TEM micrograph of a cross‐section of a) *C. albicans* K1 strain and b) *C. neoformans* H99 strain before and after PDAP_20_ Treatment. c,d) SEM images of c) *C. albicans* K1 strain and d) *C. neoformans* H99 strain before and after PDAP_20_ Treatment. e) Fluorescence confocal images demonstrate the fungicidal process. Blue and red fluorescence represent CPM‐PDAP_20_ enrichment and propidium iodide (PI) uptake, respectively.

The insusceptibility of PDAP_20_ to antifungal resistance encouraged us to explore its antifungal mechanism. We chose four folds the MIC (4 × MIC) for all antifungal mechanism tests to ensure observation of the fungal killing. Different from the standard MIC test results, the MIC of the PDAP_20_ at a high fungal cell concentration of 1.0 × 10^8^ CFU mL^−1^ (used for transmission electron microscope assay) and 3.0 × 10^6^ CFU mL^−1^ (used for other mechanism experiments) are 50 and 12.5 µg mL^−1^, respectively. For these antifungal mechanism experiments, a high concentration of fungal cells were used to facilitate the sample preparation or microscopic observation. After incubation with PDAP_20_, both *C. albicans* and *C. neoformans* showed cell lysis and large empty space in the cytosol, as well as disorganization of cytoplasm in transmission electron microscope (TEM) characterization (**Figure** **4**a,b; Figure S4, Supporting Information). This observation is echoed by the scanning electron microscope (SEM) characterization that showed severe membrane deformation and invagination after *C. albicans* and *C. neoformans* were treated with PDAP_20_ (Figure 4c,d).

We did the time‐kill assay using 2 × MFC of polymer concentration corresponding to relevant fungal cell concentration. At a fungal cell concentration of 1250 CFU mL^−1^ (for standard MIC/MFC test), 2 × MFC is 3.1 µg mL^−1^. At the fungal cell concentration of 3.0 × 10^6^ CFU mL^−1^ (used for mechanism studies), 2 × MFC is 50 µg mL^−1^. The time‐kill kinetics of PDAP_20_ agaist *C. albicans* showed that PDAP_20_ usually takes several hours to completely kill the fungi, and the time‐kill kinetics is faster at the high fungal cell concentration condition than that at the low fungal cell concentration condition (Figure S5, Supporting Information). It's worth mentioning that at the high fungal cell concentration condition, a higher concentration of the polymer PDAP_20_ was also used relative to that at the low fungal cell concentration condition and, therefore, reasonable to have a faster fungal cell kill‐kinetics. The killing efficacy is obviously dependent on the polymer concentration.

In addition, we monitored the fungicidal process of PDAP_20_ upon *C. albicans* by laser scanning confocal microscope, using 7‐diethylamino‐3‐(4‐maleimidophenyl)‐4‐methylcoumarin (CPM) conjugated PDAP_20_ to track polymer in the blue channel, and propidium iodide (PI) to detect the integrity of cell membrane in the red channel.^[^ ^16^ ^]^ The tracking started immediately after the antifungal polymer and PI were incubated with *C. albicans* cells (0 min), and images were collected every 10 min. We observed the antifungal polymer enriched on the cell membrane and formed a distinct blue ring within 10 min (Figure 4e). This phenomenon is consistent to our design of PDAPs that have higher density of positive charge than does *ε*‐PL, and are expected to have increased electrostatic interaction with fungal cell membrane. After 30–40 min, the antifungal polymer entered fungal cells but accompanied with little PI signal inside the cells. Starting from 50 to 60 min, obvious PI uptake was observed, indicating damage of cell membrane integrity by PDAP_20_. These observations indicate that PDAP_20_ enriched on fungal cell membrane first and then entered fungal cells without damaging the membrane. Notably, fungal cell membrane damage happened after PDAP_20_ was uptaken into fungal cytoplasm.

**Figure 5:**
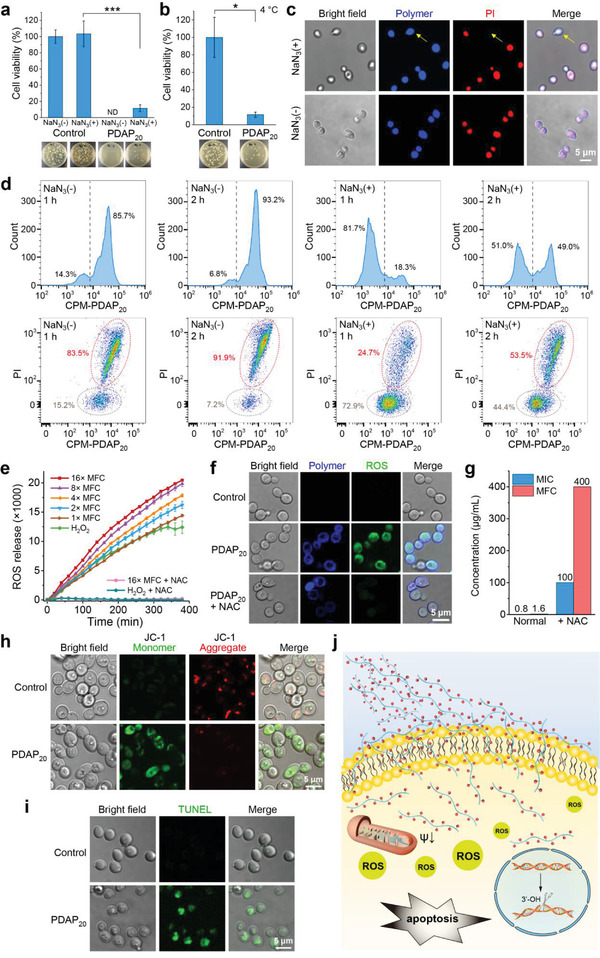
PDAP shows cell penetration, ROS generation and apoptosis of fungal cells. a,b) Killing efficiency assay under energy depletion state using a) 5 × 10^−3^ m sodium azide or b) standing at 4°C environment. *n* = 3, mean values ± s.d. Statistical analysis: two‐tailed t‐test, *p < 0.01, ***p < 0.0001. c) Colocalization of CPM‐PDAP_20_ (blue) and PI (red) fluorescence within *C. albicans* cells in the absence or presence of sodium azide. d) Flow cytometry analysis counting fluorescent fungal cells after cell incubation with 50 µg mL^−1^ CPM‐PDAP_20_ for 1 and 2 h in the presence or absence of NaN_3_. A final concentration of PI at 10 µg mL^−1^ was used for two color flow cytometry. e) ROS generation over time measured by DCFH fluorescence under different PDAP_20_ concentration with or without the ROS scavenger NAC. f) Colocalization of PDAP_20_ and DCF fluorescence (green, generated by ROS). g) Effects of ROS scavenger NAC on the inhibition and killing of *C. albicans* by PDAP_20_. h) Mitochondrial membrane potential detected with the fluorescent dye JC‐1, which exists as aggregates (red) in normal cells and as monomers (green) in apoptotic cells. i) DNA fragmentation was visualized by green fluorescence using TUNEL staining. j) Schematic diagram of proposed fungicidal mechanism for PDAP_20_.

We further explored how PDAP_20_ undergo transmembrane into fungal cells and whether this process was energy dependent. The antifungal efficacy of PDAP_20_ was tested at a concentration of 2 × MFC in the presence of 5 × 10^−3^ m NaN_3_ or at 4°C to keep fungal cells at an energy depletion condition.^[^ ^17^ ^]^ The results showed about 88% of fungal cells were still killed under both conditions (**Figure** **5**a,b), indicating that transmembrane and uptake of PDAP_20_ can occur in an energy‐independent pathway. Using confocal fluorescence microscopy to monitor the interaction between CPM‐conjugated PDAP_20_ and *C. albicans*, we observed that the antifungal polymer uptake by fungal cells happened even in the presence of NaN_3_ (Figure 5c), which echoed energy‐independent transmembrane and uptake of PDAP_20_ as observed in above antifungal efficacy study. Precedent studies on the mechanism of cell‐penetrating peptides (CPPs) provided several mechanisms for the transmembrane of CPPs.^[^ ^18^ ^]^ As the energy independent route, the transmembrane mechanism of PDAP might follow the “inverted micelle model” or the “toroidal pore formation model”.

We further did flow cytometry experiment to investigate the antifungal mechanism using fluorescent polymer (CPM‐PDAP_20_) in the presence or absence of NaN_3_. The results showed that a large proportion of fungal cells were killed in the absence of NaN_3_ (85.7% and 93.2% of total fungal cells have uptake of polymer, and 83.5% and 91.9% of total fungal cells have uptake of PI after incubation for 1 and 2 h, respectively) (Figure 5d). It is note that the polymer can still be uptaken by the fungal cells in the presence of NaN_3_ (non‐energy dependent conditions), though the transmembrane efficiency and fungicidal activity were weakened (only 18.3% and 49.0% uptake of polymer, and 24.7% and 53.5% uptake of PI after incubation for 1 and 2 h, respectively) (Figure 5d). These results indicated that in an energy‐depletion condition, PDAP_20_ can still cross the fungal membrane and get into fungal cells to kill fungi but with a reduced killing kinetics; whereas, in an energy dependent condition the transmembrane and uptake of PDAP_20_ improved significantly to have a faster killing of fungal cells. This means both the energy dependent and energy independent mechanism exist in this system.

To figure out what happened after PDAP uptake into fungal cells and what is the primary reason for fungal membrane damage, we turned our attention to intracellular reactive oxygen species (ROS) that are usually associated with the fungicidal process and cause lipid peroxidation, membrane damage of fungal cells and apoptosis.^[^ ^19^ ^]^ Using 2,7‐dichlorofluorescein (DCF) as the ROS indicator,^[^ ^20^ ^]^ we found ROS production and a gradual increase of ROS level over time within fungal cells after PDAP_20_ treatment (Figure 5e). In addition, the intracellular ROS level of PDAP_20_‐treated fungal cells was higher than that of the hydrogen peroxide‐treated fungal cells. Notably, the intracellular ROS level was dependent on PDAP_20_ concentration and increased incrementally with the increase of PDAP_20_ concentration. The intracellular ROS accumulation was further visualized and confirmed by fluorescence confocal microscopy (Figure 5f). To figure out whether ROS production is the primary reason for fungal membrane damage by PDAP_20_, we examined the antifungal activity of PDAP_20_ in the presence of 20 × 10^−3^ m NAC (antioxidant, as a ROS quencher) without affecting the growth of fungal cells (Figure 5g). We observed a remarkably reduced antifungal activity with MIC and MFC values decreased, respectively, from 0.8 to 100 µg mL^−1^ and from 1.6 to 400 µg mL^−1^, which indicated that intracellular ROS generation plays a significant role in the fungicidal activity of PDAP_20_.

ROS have been regarded as primary cell death regulators and are connected to many crucial steps of the apoptotic pathway in yeast.^[^ ^19b,d^ ^]^ The loss of mitochondrial membrane potential represents the early stage of apoptosis pathway, which can be detected by 5,5’,6,6’‐tetrachloro‐1,1’,3,3’‐tetraethylbenzimidazol‐carbocyanine iodide (JC‐1) stain showing green fluorescence.^[^ ^21^ ^]^ Our JC‐1 stain study showed that normal cells have cytosol red fluorescence; whereas, PDAP_20_‐treated fungal cells have cytosol green fluorescence, which indicates the early stage of apoptosis (Figure 5h). In addition, we analyzed DNA fragmentation of PDAP_20_‐treated fungal cells using a TdT‐mediated dUTP Nick‐End Labeling (TUNEL) assay, as a late apoptosis marker.^[^ ^22^ ^]^ The strong green cytosol fluorescence in PDAP_20_‐treated fungal cells indicated a late stage of apoptosis (Figure 5i). These fluorescence images and aforementioned electron microscope characterization altogether imply an antifungal mechanism of the highly positively charged PDAP_20_ that the polymer enriches onto the negatively charged fungal membrane first and then penetrates into fungal cells, resulting in cytosol ROS production, cell apoptosis and cell death (Figure 5j).

## 4 PDAP Effectively Inhibits the Formation of Fungal Biofilms and Eradicates the Mature Biofilms

**Figure 6:**
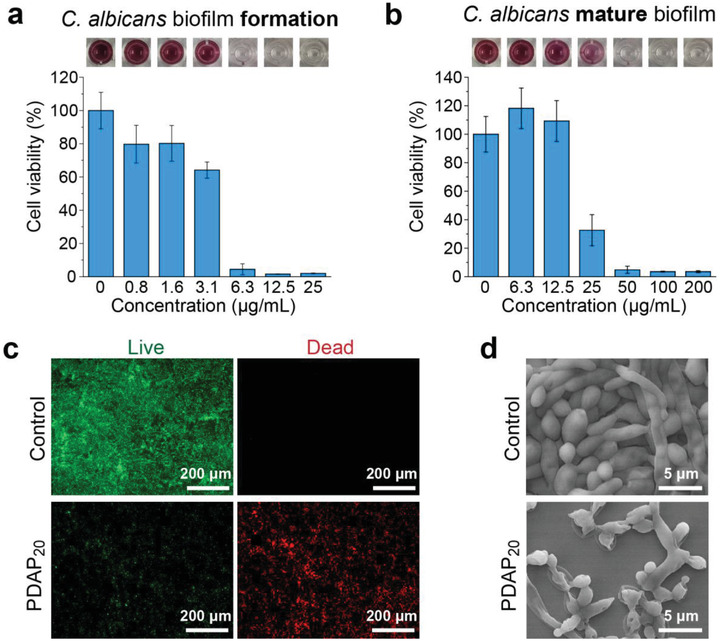
PDAP resists biofilm formation and eradicates mature biofilms. a,b) Activity of PDAP_20_ against a) *C. albicans* biofilm formation and b) mature biofilm. Embed images in (a) and (b) corresponding to biofilms that were treated with gradient concentration of PDAP_20_ and then were stained with MTT and dissolved in DMSO. c) Live/Dead fluorescence micrographs and d) SEM images of *C. albicans* mature biofilm treated with PDAP_20_ at concentrations of SMIC_80_. Untreated biofilms were used as controls.

Fungal biofilms are frequently encountered in clinical infections and are considered as a formidable challenge to have even over 1000‐folds antifugnal resistance.^[^ ^23^ ^]^ The mechanism studies on PDAP_20_ revealed that PDAP can cross the membrane to enter fungal cells and induce apoptois of fungi. PDAPs were not only fungistatic but also fungicidal, and didn't induce fungi to develop antifungal resistance. Therefore, we speculated that PDAP can still be active against mature fungal biofilms. We found that PDAP_20_ resists the formation of *C. albicans* biofilm at 6.3 µg mL^−1^ (**Figure** **6**a), and can even effectively eradicate mature *C. albicans* biofilms at 50 µg mL^−1^ (Figure 6b). Live/Dead staining indicated that PDAP_20_ effectively kill fungal cells within mature *C. albicans* biofilms (Figure 6c). SEM images showed that the untreated biofilm consisted of both oval planktonic and long tubular hyphae, while PDAP_20_ treated biofilm was destroyed (Figure 6d). The effective anti‐biofilm properties imply that PDAP_20_ is promising for clinical application.

## 5 In Vivo Efficacy of PDAP

**Figure 7:**
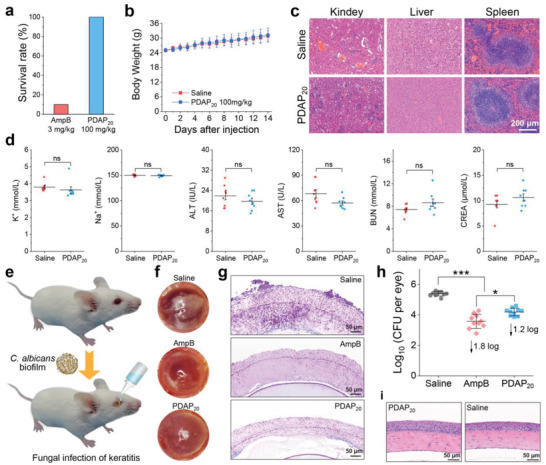
In vivo antifungal assay. a) Survival rate of male ICR mice (*n* = 10) after a single intravenous administration of AmpB and PDAP_20_ at a dose of 3 mg kg^−1^ and 100 mg kg^−1^, respectively, for 14 days. b) Daily body weight change of male ICR mice (*n* = 10, mean values ± s.d.) within 14 days after a single intravenous administration of saline or PDAP_20_ at a dose of 100 mg kg^−1^. c) H&E staining of kidney, liver, and spleen (*n* = 2) and d) serum biochemical indicators (*n* = 8, mean values ± s.d.) after a single intravenous administration of saline or PDAP_20_ at a dose of 100 mg kg^−1^. e) Schematic diagram of fungal keratitis model. f) Photograph and g) histological sections (PAS stain, fungal in purple) of representative male BALB/c mice eyes with keratitis after treatment with saline, AmpB (1 mg mL^−1^), and PDAP_20_ (15 mg mL^−1^). h) CFU of *C. albicans* SC5314 in mouse cornea treated with saline, AmpB and PDAP_20_. *n* = 10, mean values ± s.d. i) Histological sections of mouse corneas with H&E staining (nucleus in blue, and cytoplasma in purple) after topical administration of PDAP_20_ (15 mg mL^−1^). Statistical analysis: One‐way analysis of variance (ANOVA) with Tukey post‐test, *p < 0.01, ***p < 0.0001. ns: not significant.

Toxicity of PDAP_20_ was evaluated by an intravenous (IV) injection of PDAP_20_ in ICR mice, using AmpB for comparison. After IV injection of a signal dose of 3 mg kg^−1^ of AmpB, 90% of the mice died within 48 h; in sharp contrast, all mice survived after IV injection of 100 mg kg^−1^ of PDAP_20_ without obvious change of mice (**Figure** **7**a), including a normal body weight compared to the saline group (Figure 7b). The main metabolic organs including kidney, liver, and spleen of the mice were sectioned and stained by H&E after IV injection of PDAP_20_ for 14 days. All these analyses showed that IV injection of PDAP_20_ resulted in no obvious toxicity on major organs (Figure 7c). Additional study on blood biochemistry showed that PDAP_20_ did not cause significant changes in blood biochemical indicators including concentration of K^+^, Na^+^, alanine aminotransferase (ALT), aspartate aminotransferase (AST), blood urea nitrogen (BUN) and creatinine (CREA) (Figure 7d), indicating the low hepatotoxicity and nephrotoxicity of PDAP_20_. All these studies showed that PDAP_20_ has low toxicity and is safe for in vivo application in treating fungal infections.

Encouraged by the potent antifungal activity in vitro and low toxicity of PDAP_20_, we continued to examine the in vivo fungicidal activity and therapeutic potential of PDAP_20_ using a biofilm‐induced fungal keratitis model (Figure 7e). After the eyeballs of male BABL/c mice were infected with *C. albicans* to have keratitis, eye ulcers were clearly observed with a dense opaque appearance. These keratitis mice were randomly grouped and treated with eye drops containing saline, AmpB and PDAP_20_, respectively. Saline‐treated mice had serious ulcers on the eyeballs, and a large amount of planktonic and filamentous fungi in the periodic acid‐schiff (PAS) stained tissue slides corresponding to 5.4 log CFU per eye (Figure 7f–h). Both AmpB and PDAP_20_ treatment remarkably alleviated eye ulcers, and significantly reduced the number of colonies by 1.8 and 1.2 log CFU per eye, respectively (Figure 7f–h). In addition, we did histological analysis on mouse corneas after PDAP_20_ treatment at above administration concentration and found no obvious toxicity (Figure 7i). Therefore, PDAP_20_ is a safe and effective antifungal agent in treating fungal keratitis.

## Conflict of Interest

The authors declare no conflict of interest.

## Author Contributions

R.L. directed the whole project. D.Z. and R.L. conceived the idea, proposed the strategy, designed the experiments, evaluated the data, and wrote the manuscript together. D.Z. performed majority of the experiments. C.S. participated in vitro and in vivo antifungal experiments. Z.C. performed the keratitis experiment. Q.C. conducted the confocal experiments and cytotoxicity assay, and drew schematic diagrams. Y.B. and J.Z. participated in the in vitro antifungal experiments. K.M., S.L., X.X., and S.C. participated the drug resistance studies. J.G. participated the cytotoxicity assay. M.C, Z.L., H.Z., L.L., W.J., and M.L. participated the in vivo experiments. J.X. operated the SEM test. N.S. participated the flow cytometry experiment. M.Z. participated in the polymer synthesis. X.S. and Y.D. isolated the fungi. L.Z. participated in result discussion and troubleshooting. All authors discussed the results and contributed to the manuscript.

## Supporting information

Supporting Information

## Data Availability

The data that support the findings of this study are available from the corresponding author upon reasonable request.
