# Supplementary material for: Microbial Metabolite Inspired β‐Peptide Polymers Displaying Potent and Selective Antifungal Activity
Source: Adv Sci (Weinh). 2022 Mar 20;9(14):2104871. doi: 10.1002/advs.202104871 (PMC9108603; doi:10.1002/advs.202104871)
Supplement: Supplementary file 1 — Supporting Information [file ADVS-9-2104871-s001.pdf]

ADVANCED SCIENCE
Open Access

Supporting Information

for *Adv. Sci.*, DOI 10.1002/advs.202104871

Microbial Metabolite Inspired *β*-Peptide Polymers Displaying Potent and Selective Antifungal Activity

*Donghui Zhang, Chao Shi, Zihao Cong, Qi Chen, Yufang Bi, Junyu Zhang, Kaiqian Ma, Shiqi Liu, Jiawei Gu, Minzhang Chen, Ziyi Lu, Haodong Zhang, Jiayang Xie, Ximian Xiao, Longqiang Liu, Weinan Jiang, Ning Shao, Sheng Chen, Min Zhou, Xiaoyan Shao, Yidong Dai, Maoquan Li, Lixin Zhang and Runhui Liu**

Supporting Information

Microbial Metabolite Inspired β-Peptide Polymers Displaying Potent and Selective Antifungal Activity

*Donghui Zhang, Chao Shi, Zihao Cong, Qi Chen, Yufang Bi, Junyu Zhang, Kaiqian Ma, Shiqi Liu, Jiawei Gu, Minzhang Chen, Ziyi Lu, Haodong Zhang, Jiayang Xie, Ximian Xiao, Longqiang Liu, Weinan Jiang, Ning Shao, Sheng Chen, Min Zhou, Xiaoyan Shao, Yidong Dai, Maoquan Li, Lixin Zhang, Runhui Liu***

Dr. D. Zhang, Dr. M. Zhou, Prof. L. Zhang, Prof. R. Liu

State Key Laboratory of Bioreactor Engineering, East China University of Science and Technology, Shanghai 200237, China

E-mail: rliu@ecust.edu.cn

C. Shi, Z. Cong, Dr. Q. Chen, Y. Bi, J. Zhang, K. Ma, S. Liu, J. Gu, M. Chen, Z. Lu, H. Zhang, J. Xie, X. Xiao, L. Liu, W. Jiang, N. Shao, S. Chen, Prof. R. Liu

Key Laboratory for Ultrafine Materials of Ministry of Education, Frontiers Science Center for Materiobiology and Dynamic Chemistry, Research Center for Biomedical Materials of Ministry of Education, School of Materials Science and Engineering, East China University of Science and Technology, Shanghai 200237, China

X. Shao, Prof. Y. Dai

Shanghai Ruijin Rehabilitation Hospital, Shanghai 200023, China

Prof. M. Li

Department of Interventional and Vascular Surgery, Shanghai Clinical Research Center for Interventional Medicine, Shanghai Tenth People's Hospital, Tongji University School of Medicine, Shanghai 200072, China

Materials. All chemical reagents and solvents were purchased from Adamas-beta® and used without further purification. *C.albicans* and *C.neoformans* were obtained from Shanghai Ruijin Rehabilitation Hospital. RPMI 1640 medium, yeast extract-peptone-dextrose (YPD), sabouraud dextrose agar (SDA), Luria Bertani (LB), Mueller-Hinton (MH) and agar was obtained from Shanghai Maokang Biotechnology Co., Ltd. NIH 3T3 fibroblast cells, endothelial cells (HUVEC), macrophage (RAW264.7), liver cells (LO2), kidney cells (COS7) and enterocyte (NCM-460) were obtained from the Cell Bank of Typical Culture Collection of Chinese Academy of Sciences (Shanghai, China). Dulbecco's modified eagle medium (DMEM) was obtained from GE Life Science (AC13298277); Fetal Bovine Serum (FBS) was obtained from Biological Industries (1707254). Information about kit is in the specific experimental sections. The water used in these experiments was obtained from a Millipore water purification system with a minimum resistivity of 18.2 MΩ. cm.

Instruments. Synthesized chemicals were purified using a SepaBean machine equipped with Sepaflash columns produced by Santai Technologies Inc. in China. ^{1}H and ^{13}C NMR spectra were collected on an AVANCE III 400 spectrometer at 400 MHz and 100 MHz, respectively, or an AVANCE III 500 spectrometer at 500 MHz and 125 MHz, respectively, using $CDCl_3$, D_2O, or DMSO-d6 as the solvent. ^{1}H NMR chemical shifts were referenced to the resonance for residual protonated solvent (δ 0.00 for TMS in $CDCl_3$, 4.79 for D_2O and 2.50 for DMSO-d6). ^{13}C NMR chemical shifts were referenced to the solvent (δ 77.16 for $CDCl_3$, 39.52 for DMSO-d6). Mass spectra were acquired using a Waters XEVO G2 TOF mass spectrometer. Gel permeation chromatography (GPC) was performed on a Waters GPC instrument equipped with a Waters 1515 isocratic HPLC pump, a Waters 2414 refractive index detector using dimethyl formamide (DMF) supplemented with 0.01 M LiBr as the mobile phase at a flow rate of 1 mL/min at 50 °C. The GPC was equipped by a Tosoh TSKgel Alpha-2500 column

(particle size 7 μm) and a Tosoh TSKgel Alpha-3000 column (particle size 7 μm) linked in series. Relative number-average molecular weight (Mn) and dispersity index (*Đ*) were calculated from a calibration curve using polymethyl methacrylate as standards. Information about other instruments and equipment is in the specific experimental part.

Monomer Synthesis.

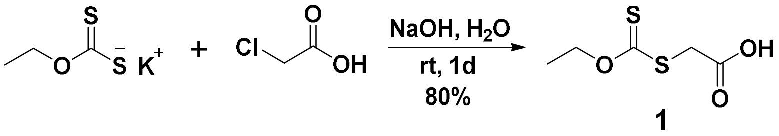

2-(Ethoxycarbonothioylthio)acetic acid (1). 2-(Ethoxycarbonothioylthio)acetic acid was synthesized by following the method in precedent literature.[1] Chloroacetic acid (50 g, 529 mmol) and sodium hydroxide (23.3 g, 582 mmol) were added to a 1000 mL round bottom flask containing 265 mL deionized water. Potassium ethlxanthate (99.8 g, 529 mmol) was added slowly to above solution, and the mixture was stirred for 1 day. Then, the reaction mixture was adjusted to pH 2 by sat. HCl in water, and the mixture was poured into CH_2Cl_2. After phase separation, the crude product was extracted into CH_2Cl_2 (150 mL × 3). The combined organic layer was washed with brine, dried over $MgSO_4$ and concentrated under reduced pressure. The residue was recrystallized from CH_2Cl_2/hexane to afford 2-(ethoxycarbonothioylthio)acetic acid **1** as a white crystal (75.6 g, 80%). ^{1}H NMR (400 MHz, $CDCl_3$) δ 11.46 (s, 1H), 4.66 (q, J = 7.1 Hz, 2H), 3.97 (s, 2H), 1.42 (t, J = 7.1 Hz, 3H). ^{13}C NMR (100 MHz, $CDCl_3$) δ 212.07, 174.61, 71.03, 37.71, 13.78. ESI-MS: $[M+H]^+$: 181.13.

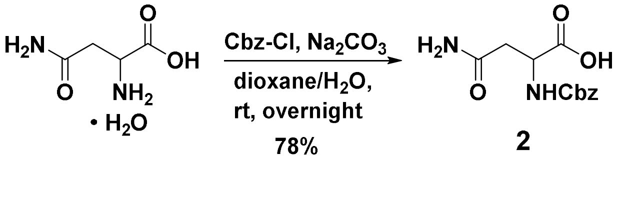

N-Carbobenzoxy-DL-asparagine (2). Compound **2** was synthesized by following the method in precedent literature.[2] DL-asparagine (40 g, 266 mmol) and sodium carbonate (56.5 g, 533 mmol) were dissolved in a mixture of deionized water (220 mL) and dioxane (110 mL), followed by the injection of benzyl chloroformate (41.2 mL, 293 mmol) at 0°C. The solution was stirred at room temperature overnight. Then, the reaction mixture was adjusted to pH 2 by sat. HCl in water. A large amount of precipitation appeared, and then the mixture was filtered. The filter cake was washed thoroughly by H_2O and diethyl ether, and dried in vacuo to afford N-Carbobenzoxy-DL-asparagine **2** as a white solid (55 g, 78%). ^{1}H NMR (400 MHz, DMSO-d6) δ 12.71 (s, 1H), 7.46 (d, J = 8.3 Hz, 1H), 7.42-7.25 (m, 6H), 7.00-6.85 (m, 1H), 5.03 (s, 2H), 4.45-4.29 (m, 1H), 2.60-2.42 (m, 2H). ^{13}C NMR (100 MHz, DMSO-d6) δ 173.28, 171.28, 155.87, 137.00, 128.41, 127.88, 127.77, 65.50, 50.67, 36.80. ESI-MS: $[M+H]^+$: 267.24, $[M+Na]^+$: 289.23.

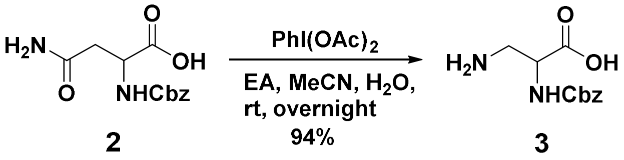

(±)-3-Amino-2-benzyloxycarbonylaminopropanoic acid (3). Compound **3** was synthesized by following the method in precedent literature.[2] To a suspension of compound **2** (55 g, 195 mmol) in a mixture of 200 mL ethyl acetate (EtOAc), 200 mL acetonitrile (MeCN) and 100 mL H_2O added iodosobenzene diacetate (69.2 g, 215 mmol) in 10 minutes. The mixture turned transparent after 1 hour of stirring and a large amount of solid separated out after stirring overnight. The resultant suspension was filtered and the filter cake was collected. The filtrate was concentrated, poured into EtOAc and filtered. The two portions of filter cake were combined and washed by EtOAc and diethyl ether thoroughly, and dried in vacuo to afford (±)-3-Amino-2-benzyloxycarbonylaminopropanoic acid **3** as white solid (46.3 g, 94%). ^{1}H NMR (400 MHz, D_2O:TFA = 5:1) δ 7.40-7.15 (m, 5H), 5.00 (s, 2H), 4.42 (dd, J = 8.8, 5.2 Hz, 1H), 3.41 (dd, J = 13.4, 5.3 Hz, 1H), 3.19 (dd, J = 13.4, 8.7 Hz, 1H). ^{13}C NMR (100 MHz, D_2O:TFA = 5:1) δ 171.66, 157.82, 135.81, 128.66, 128.38, 127.70, 67.43, 51.36, 39.64. ESI-MS: $[M+H]^+$: 239.25, $[M+Na]^+$: 261.21.

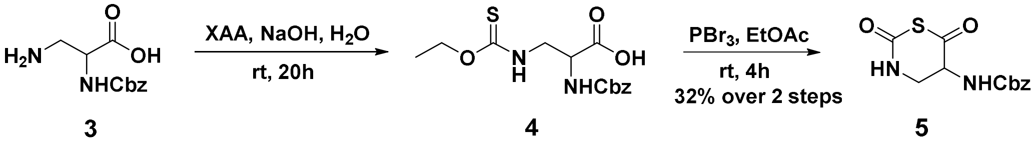

Synthesis of Cbz-β^2-DL-DAP (5). Compound **5** was synthesized by following the method in precedent literature.[1] To a solution of compound **3** (10.0 g, 42.0 mmol) and sodium hydroxide (3.53 g, 88.2 mmol) in deionized water (84 mL) added compound **1** (7.57 g, 42.0 mmol). The mixture was stirred at room temperature for 20 h, and then adjusted pH to 2 by 2 N HCl. The resultant was extracted by EtOAc three times, and the combined organic layer was washed with brine, dried over $MgSO_4$ and concentrated. The residue was recrystallized

from CH_2Cl_2 to afford 13.52 g the crude compound **4** as a white solid and was used in the next step without further purification. In a 250 mL pre-dried round flask, phosphorus tribromide (0.84 mL, 8.80 mmol) was dropwise injected to a suspension of crude compound **4** (2.45 g, 7.33 mmol) in anhydrous EtOAc (37 mL) at 0 °C under N_2 atmosphere. The mixture was stirred at room temperature for 4 hours, and then quenched by cold deionized water (30 mL). After phase separation, the organic layer was washed with cold deionized water (20 mL × 3) and brine, dried over $MgSO_4$ and concentrated in vacuo. The residue was recrystallized by EtOAc and petroleum ether to afford **5** as white floccule (694 mg, 32% over 2 steps). ^{1}H NMR (500 MHz, $CDCl_3$:DMSO-d6= 2:1) δ 9.01 (d, J = 5.9 Hz, 1H), 7.58 (d, J = 8.3 Hz, 1H), 7.40-7.28 (m, 5H), 5.10 (s, 2H), 4.48 (q, J = 3.7 Hz, 1H), 3.57 (t, J = 12.6 Hz, 1H), 3.39 (dt, J = 13.0, 5.4 Hz, 1H). ^{13}C NMR (125 MHz, $CDCl_3$:DMSO-d6 = 2:1) δ 194.93, 162.45, 155.68, 135.83, 127.90, 127.55, 127.45, 66.12, 56.92, 41.01. HRESI-MS m/z calcd for $C_{12}H_{12}N_2NaO_4S$ $[M+Na]^+$: 303.0415, found 303.0416.

Synthesis of PDAP.

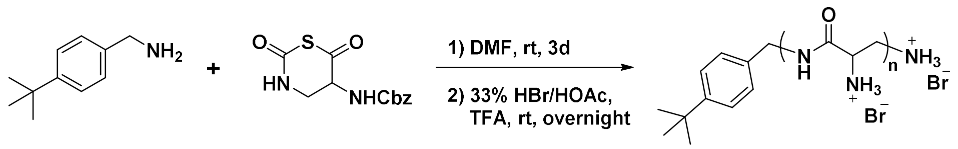

Polymers were synthesized using previously reported methods.[1] Cbz-β^2-DLDAP (56 mg, 0.2 mmol) was weighed out and added to a glass vial at room temperature. To the vial was added anhydrous DMF to a final monomer concentration of 0.5 M. The polymerization was initiated by the addition of a solution of 0.22 M 4-tert-butylbenzylamine in DMF. The polymer length was controlled by the molar ratio of monomer to the initiator. The reaction mixture was stirred at room temperature for 3 days and then the resulting polymer was precipitated by pouring into 2 mL tetrahydrofuran (THF) and 45 mL petroleum ether. The solid was isolated by centrifugation, and the supernatant liquid was decanted off. The solid was re-dissolved in

THF and re-precipitated with petroleum ether. After three repetitions of the precipitation/centrifugation procedure, the white pellet of Cbz-protected PDAP was collected and dried under vacuum.

Deprotection of the polymer was achieved by dissolving the white solid in 1 mL of trifluoroacetic acid and 1 mL of 33% HBr in HOAc. The reaction mixture was shaken overnight at room temperature. The resulting solution was partially volatilized by air and poured into cold diethyl ether, the resulting precipitate was isolated by centrifugation, and the supernatant liquid was decanted off. The solid was dried under a stream of air. After two more repetitions of the precipitation/centrifugation procedure by methanol/ether, the white pellet was collected and dried under vacuum. The material was then dissolved in approximately 6 mL of water, filtered through 0.45 μm polyether sulfone membrane and lyophilized. After side chain deprotection, the degree of polymerization (DP) was characterized by ^{1}H-NMR using D_2O as the solvent. For polymer stability test, $PDAP_{20}$ were dissovled in saturated NaCl, 0.1 N HCl and 0.1 M NaOH for 24 h, respectively. Then acid or base treated polymers were neutralize using 0.1 M NaOH and 0.1 N HCl. The treated polymers were freezing-dried and characterized by ^{1}H-NMR using D_2O as the solvent.

Synthesis of 7-diethylamino-3-(4maleimidophenyl)-4-ethylcoumarin labelled PDAP.

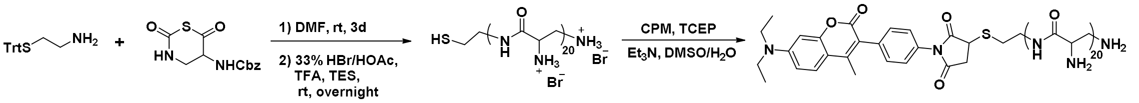

Synthesis of thiol-ended PDAP follows the same procedure as above mentioned tBuBz-ended polymers. For conjugating with 7-diethylamino-3-(4maleimidophenyl)-4-ethylcoumarin (CPM), thiol-ended PDAP (3 mg, 0.86 μmol), CPM (0.7 mg, 1.71 μmol) and tris(2-carboxyethyl)phosphine (TCEP) (0.3 mg, 0.86 μmol) were dissolved in 600 μL DMSO:H_2O = 1:1, and then 20 μL triethylamine (Et_3N) was added to the mixture. The resultant mixture was shaken for 2 hours in dark, and poured into 15 mL H_2O to precipitate excess CPM. After

centrifuge, the supernatant was filtered through 0.22 μm nylon membrane and freeze-dried. The obtained crude product was washed with 50 mL acetone twice, 50 mL diethyl ether once and dried in vacuo. The product was dissolved in 2 mL H_2O, filtered through 0.22 μm polyethersulfone membrane and lyophilization to give 1.8 mg of the product (95% yield) as a light green solid. HPLC assay indicates no CPM residue in the product.

In vitro antifungal assays. The MIC assays for *C. albicans* (K1, SC5314, Gu5, ATCC90028, R01, R02, R03 and R04 strains) and *C. neoformans* (H99, MYA737 and JEC21 strains) were conducted according to a previously described protocol.[3] The cultured fungal cells were collected at 2.5×10^3 CFU/mL in RPMI 1640 medium to give the working suspension. Two-fold serial dilution of PDAP, AmpB and fluconazole in RPMI was conducted in a 96-well plate to give 50 μL compound solution in each well. Then 50 μL of the cell suspension was added to each well. Wells containing cells in RPMI without any polymer or antifungal drug were used as drug-free controls, and wells containing RPMI medium only were used as cell-free controls. The plate was gently stirred for 10 seconds and then incubated at 30 °C. After *C. albicans* and *C. neoformans* cells were incubated for 24 h and 48 h, respectively, fungal cell growth was checked visually. The MIC values were identified as the lowest concentration of an antifungal agent to completely inhibit fungal cell growth, that is, no cell growth was visible in the well. MIC_{50} was used for fluconazole because it cannot inhibit 100% growth of *C. albicans*. The MFC assay for *Candida* and *Cryptococcus* was conducted after the MIC test. An aliquot of 3 μL premixed suspension from each well was plated on the yeast extract-peptone-dextrose (YPD) agar. The plates were incubated at 30 °C for 24 h and 48 h, respectively, for *C. albicans* cells and *C. neoformans* cells. Then the plate was inspected visually for colony formation. The MFC values were identified as the lowest concentration to

kill all (> 99.9%) of the cells, which means no fungal cell colony was observed. The antifungal assays were performed at least three times at different time.

The antimicrobial activities of $PDAP_{20}$ and ε-PL were also tested similar to the aforementioned antifungal assay. Gram-positive bacteria (*S. aureus* ATCC 6538, *S. epidermidis* ATCC 49134, *B. subtilis* BR151) and Gram-negative bacteria (*E. coli* ATCC 25922, *A. Baumannii* ATCC 747, *K. pneumoniae* ATCC 700603) were used in the test. Bacteria were cultured by LB growth medium, and the bacterial concentration of 1.0×10^5 CFU/mL in MH growth medium was used for antibacterial test. The positive controls used in Gram-positive and Gram-negative bacteria are vancomycin and polymyxin B, respectively.

Hemolysis assay. Hemolysis assays were conducted as previously described using human red blood cells (RBCs).[4] Human blood (5 mL) was washed three times with Tris-buffered saline (TBS, pH 7.2) containing 10 mM Tris and 150 mM NaCl. The collected RBCs were suspended in TBS (250 mL) to obtain a working suspension of 5% RBC relative to total RBCs in the whole blood. Two-fold serial dilution of PDAP was conducted in a 96-well plate in TBS to obtain concentrations ranging from 2000 to 15.6 μg/mL. Each sample well had 100 μL of compound solution after the 2-fold serial dilution. Then, 100 μL of the RBC working suspension was added to each well, followed by gentle shaking of the plate for 10 s. On the same plate, wells containing TBS without polymer were used as the blank; wells containing Triton X-100 (3.2 mg/mL in TBS) were used as the positive control. The plate was incubated at 37 °C for 1 h and then centrifuged at 3700 rpm for 5 min to precipitate the RBCs. An aliquot of 80 μL of the supernatant from each well was transferred to the corresponding well in a new 96-well plate, and the optical density (OD) at 405 nm was measured using a Molecular Devices Emax precision microplate reader. Measurements were performed in duplicate and repeated on three different days. The percentage of hemolysis at each polymer concentration was calculated from [% hemolysis = $100 \times (A_{polymer} - A_{blank})/(A_{control} - A_{blank})$]

and plotted against polymer concentration to give the dose-response curves for hemolysis for each polymer. The HC_{10} value for each polymer was defined as the polymer concentration to cause 10% lysis of RBCs. All measurements were performed with three replicates.

Cytotoxicity assay. Cytotoxicity tests were performed on NIH 3T3 fibroblast cells, endothelial cells (HUVEC), macrophage (RAW264.7), liver cells (LO2), kidney cells (COS7) and enterocyte (NCM-460) using MTT assay.[3] Cells were cultured in DMEM supplemented with 10% FBS, 5% penicillin, 2 mM L-glutamine and incubated at 37 °C in 5% CO_2. Cells were harvested and seeded onto 96-well plates at a density of 1.0×10^4 cells per well and incubated for 24 h. On each plate, wells containing growth medium only and cells in growth medium without any antifungal agent were used as the negative and positive control, respectively. PDAP or AmpB were diluted with the growth medium to give final concentrations ranging from 800 to 1.6 μg/mL. Medium in each cell-containing well was replaced with 100 μL of the corresponding polymer solution, and the plates were incubated at 37 °C for 24 h. Then, the solution in each well was switched to 100 μL MTT solution (1 mg/mL) and the plate was incubated in the dark at 37 °C for 4 h. After centrifuge (to prevent the possible loss of formazan crystals) and removal of the supernatant, 100 μL DMSO was added to each well to dissolve the formed formazan under stirred for 20 min. Then 85 μL of the supernatant from each well was transferred to the corresponding well in a new plate, followed by absorbance measurement at 570 nm on a plate reader. Each antifungal agent was tested in triplicate in each assay, and the experiment was conducted at least twice on a different day. The percentage of cell viability in each test well, relative to the positive control (which defines 100%) w as calculated from [% fibroblast growth = $(A_{polymer} - A_{blank})/(A_{control} - A_{blank}) \times 100$]. The IC_{50} value is the minimum concentration to inhibit 50% of fibroblast cell growth. All measurements were performed with three replicates.

Drug resistance studies. Drug resistance was induced by treating the *C. albicans* (R02 strain) repeatedly with antifungal agents. The MIC of $PDAP_{20}$ and MIC_{50} of fluconazole against *C. albicans* were tested for 30 passages of growth, which is long enough and adopted by many studies.[5] *C. albicans* exposed to the 1/2 MIC concentration at the particular passage were re-grown to a logarithmic growth phrase and re-used for the subsequent passage's MIC measurement for the same antifungal agent. Drug-resistant behavior of *C. albicans* was evaluated by recording the changes in the MIC compared to the original passage. All measurements were performed in duplicates.

Kill kinetics assay. A culture of *C. albicans* K1 strain was collected at 2.5×10^3 CFU/mL or 6.0×10^6 CFU/mL in RPMI 1640 medium to give the working suspension. 0.5 mL of $PDAP_{20}$ solution was added to 0.5 mL of the above fungal suspension in each well of a 24-well plate to achieve 2 × MFC of polymer concentration. The fungal suspensions were incubated at 30 °C. At desired time points, each suspension was homogeneously mixed and 30 μL of each sample was serial diluted in PBS, and plated on YPD agar plates for CFU determination. All measurements were performed in duplicates.

SEM analysis. *C. albicans* (K1 strain) and *C. neoformans* (H99 strain) cells (3.0×10^6 CFU/mL) before and after incubation with $PDAP_{20}$ at 50 μg/mL (2 × MFC concentration) for 4 h were harvested by centrifugation at 4000 rpm for 5 min. The collections were washed by PBS three times and then fixed in 4% glutaraldehyde solution overnight at 4 °C. Cells were further washed with Millipore water, followed by dehydration using a series of ethanol solutions with different volume contents (35, 50, 75, 90, 95 and 100%). The sample was then placed on a gold-coated quartz chip and further sprayed with gold. The morphologies of the fungi before and after polymer treatment were observed using a field emission scanning electron microscope (S-4800, Hitachi Limited).

TEM analysis. *C. albicans* K1 strains and *C. neoformans* H99 strains (1.0 × 10^8 CFU/mL, 4 mL) before and after incubation with $PDAP_{20}$ at 200 μg/mL for 4 h were harvested by centrifugation at 4000 rpm for 5 min. The collections were washed by PBS three times, and then fixed in 2.5% glutaraldehyde solution overnight at 4 °C. The supernatants were decanted, and the fixed fungal cells were washed with phosphate buffer (PB, 0.1 M, pH = 7.0) for three times (15 min each), and post-fixed with 1% OsO_4 in the PB for 1 h. After the supernatants were carefully removed, the fixed samples were washed with PB three times (15 min each), followed by dehydration using a series of ethanol solutions and finally treated with pure ethanol and acetone (20 min each). The samples were incubated with a mixture of acetone and Spurr resin (1:1 in volume) for 1 h and then transferred to a 1:3 mixture of acetone and Spurr resin for another 3 h incubation, followed by final incubation in Spurr resin overnight. Ultrathin sections (70-90 nm) were obtained with a LEICA EM UC7 ultramicrotome and poststained with lead citrate and 50% uranyl acetate/EtOH for 10 min. The morphologies of the fungi before and after polymer treatment were observed using a transmission electron microscope (120 kV, H-7650, Hitachi).

Real-time monitoring of antifungal process. An aliquot of 90 μL *C. albicans* (K1 strain) cell suspension at 3.0 × 10^6 CFU/mL was added to a confocal dish and kept still for 30 min for settlement. Then a random area of the dish was locked and the focal length was adjusted under 100× oil immersion lenses, with further fourfold electronic amplification. The images of bright field, blue and red channel were collected and defined as 0 min. Then 5 μL propidium iodide solution (PI, 200 μg/mL in PBS) and 5 μL CPM-$PDAP_{20}$ solution (1 mg/mL in H_2O, for finally 2 × MFC at this cell concentration) were gently pipetted to the fungal solution and mixed well. Then, images were gathered every 10 min until all cells in this sight were killed.

Energy depletion analysis. For energy depletion experiments, cell suspension of *C. albicans* (K1 strain) at 3.0 × 10^6 CFU/mL was pretreated with RPMI containing 5 mM NaN_3 for 1 h.[6]

For antifungal efficiency assay, a fresh cell suspension pretreated or non-pretreated with NaN_3 were incubated with 50 μg/mL $PDAP_{20}$ or without an antifungal agent (as energy depletion control) for 24 h. The fresh cell suspension without antifungal agents before and after incubation with NaN_3 for 24 h were used as the controls, respectively. The suspensions were diluted and plated on YPD agar. The plates were incubated at 30 °C for 24 h and colony amounts were counted. The cell viability after incubation at 4 °C with 50 μg/mL $PDAP_{20}$ or without an antifungal agent (as positive control) was measured using the same method.

For the confocal assay, a fresh cell suspension pretreated or non-pretreated with NaN_3 was incubated with 50 μg/mL CPM-$PDAP_{20}$ for 24 h, respectively. The resultant suspensions were spread on the confocal dishes and incubated with 10 μg/mL PI for 10 min. The images of bright field, blue and red channel were collected under 100× oil immersion lenses. Representative areas in the images were electronically amplified fourfold.

For flow cytometry experiment, a fresh cell suspension pretreated or non-pretreated with NaN_3 was incubated with 50 μg/mL CPM-$PDAP_{20}$ for 1 h and 2 h, respectively. Fungal cells were washed by PBS to remove polymers, and 5.0 × 10^4 cells were counted and anaysized. The polymer uptake was calculated by flow cytometry (Backman). Furthermore, cell suspensions were incubated with 10 μg/mL PI for 10 min for calculated PI uptake.

Intracellular reactive oxygen species (ROS) detection and relation to antifungal activity. ROS assays were conducted using a method similar to reported protocol.[6-7] Six columns of two-fold serial dilution of $PDAP_{20}$ (from 200 to 1.6 μg/mL) were prepared in a 96-well plate using RPMI to give 50 μL polymer solution in each well, with three columns containing normal RPMI and other three columns containing RPMI supplemented with 20 mM NAC,[8] a

NAC concentration without affecting the normal cell growth. Wells containing cells in RPMI and 40 mM H_2O_2 was used as positive controls. Cell suspension of *C. albicans* (K1 strain) at 6.0×10^6 CFU/mL was treated with 20 μM 2,7-dichlorodi-hydrofluorescein diacetate (DCFHDA) for 20 min. Then 50 μL of the cell suspension was added to each well, except the blank control (RMPI containing DCFHDA without cells). The fluorescence intensity of the induced ROS production was measured every 30 min using a plate reader at λ_{ex} = 485 nm and λ_{em} = 530 nm. The confocal observation of ROS production was conducted after *C. albicans* (K1 strain) suspension at 3.0×10^6 CFU/mL was incubated with 50 μg/mL CPM-$PDAP_{20}$ for 2 h. The images of bright field, blue and green channel were collected under 100× oil immersion lenses, with representative areas in the images electronically amplified sixfold.

Detection of mitochondrial membrane potential. Changes in mitochondrial membrane potential in *C. albicans* after incubation with $PDAP_{20}$ were analyzed using JC-1 as previously described.[9] Mitochondrial membrane potential was detected with the fluorescent dye JC-1, which exists as aggregates (red) in normal cells with polarized mitochondria, whereas exists as monomers (green) in apoptotic cells with depolarized mitochondria. An aliquot of 0.8 mL *C. albicans* (K1 strain) suspension at 3.0×10^6 CFU/mL was incubated with 50 μg/mL $PDAP_{20}$ for 2 h, followed by the procedure of using JC-1 kit (Solarbio, M8650). A fresh non-treated cell suspension was used as the control. After staining and washing steps, the cells were spread on a confocal dish. Images of bright field, green (JC-1 aggregation) and red (JC-1 monomer) channels were collected under 100× oil immersion lenses, with representative areas in the images electronically amplified sixfold.

DNA fragmentation assay. Apoptosis induced DNA fragments in *C. albicans* cells were analyzed using the TdT-mediated dUTP Nick-End Labeling (TUNEL) method.[10] An aliquot of 0.8 mL *C. albicans* (K1 strain) suspension at 3.0×10^6 CFU/mL was incubated with 50

μg/mL $PDAP_{20}$ for 8 h, followed by the procedure of using TUNEL kit (Beyotime, C1086). A fresh non-treated cell suspension was used as a control. The bright field and FITC fluorescence were collected under 100× oil immersion lenses, with representative areas in the images electronically amplified sixfold.

Anti-biofilms. This study was conducted in 96-well plates (flat bottom and tissue-culture treated) by following the reported protocol with slight modifications.[4, 11] On each plate, wells containing RPMI only and cells in medium without any antifungal agent were used as the negative and positive control, respectively.

For **biofilm formation:** Freshly cultured fungal cells were collected to prepare the working suspension at 1.0 × 10^6 CFU/mL in RPMI. A two-fold serial dilution for each antifungal agent was prepared using a corresponding medium in a 96-well plate to give 100 μL of polymer solution in each well. 100 μL of the working suspension was added to each well except the controls, and the plate was incubated at 37 °C for 48 h. Afterward, the solution in each well was removed and each well was washed with 200 μL PBS three times, followed by addition of 100 μL MTT solution (1 mg/mL). The MTT assay protocol was the same as the above mentioned protocol in fibroblast cell toxicity assay. This value was calculated from [% biofilm growth = $(A_{polymer} - A_{blank})/(A_{control} - A_{blank}) \times 100$] and plotted against polymer concentration to give the dose-response graphs for inhibition of biofilm formation for each antifungal agent. The $SMIC_{80}$ value is the minimum concentration of a given agent that is necessary to inhibit 80% of fungal biofilm formation.

For **mature biofilms:** Freshly cultured fungal cells were collected to prepare the working suspension at 5.0 × 10^5 CFU/mL in RPMI. 200 μL of this cell working suspension was added to each well of a 96 well plate (flat bottom and tissue-culture treated) except the blank controls and the plate was incubated at 37 °C for 48 h in a moisture-controlled incubator.

After the old medium was removed, each well was washed with 200 μL PBS three times followed by the addition of 150 μL RPMI to each well. 150 μL solution of the antifungal agent at gradient concentration was added to the corresponding well of the 96-well plate containing fungal biofilms, and then the plate was incubated at 37 °C for 48 h. The $SMIC_{80}$ value of antifungal agents eliminating mature biofilms was measured by MTT assay using aforementioned protocol in the biofilms formation assay.

Live/Dead staining and SEM imaging of biofilms. Mature *C. albicans* (K1 strain) biofilms were formed after 48 h culture of planktonic cells in a 96-well plate (for Live/Dead staining) and a 24-well plate contains gold-coated chips (for SEM imaging) as described above. The medium in each well was carefully removed, and these biofilms were treated with $PDAP_{20}$ in RPMI at a concentration of $SMIC_{80}$ value (50 μg/mL) or without an antifungal agent (as a control) at 37 °C for 48 h. The medium was removed from each well and the biofilm in each well of the 96-well plate was washed three times with 100 μL PBS and then incubated with 40 μL of Live/Dead dye solution in PBS (Thermo Fisher Scientific, L13152. Containing 6 μM SYTO 9 and 30 μM propidium iodide) at room temperature for 15 min. The biofilm in each well was then washed three times with 100 μL PBS, and then fluorescent images of the biofilms were captured using a fluorescence microscope. The biofilms on the surface of gold chips were washed three times with 1 mL PBS and then were fixed with 2.5% glutaraldehyde overnight at 4 ºC. The fixed platelets were dehydrated with a series of ethanol solutions (35%, 50%, 75%, 90%, 95% and 100%, 15 min each). SEM images of biofilms before and after $PDAP_{20}$ treatment were collected on a field emission SEM (S-4800, Hitachi Limited).

In vivo acute toxicity. All animal procedures were performed in accordance with the Guidelines for Care and Use of Laboratory Animals of the Shanghai Tenth People's Hospital

(License number: SYXK(沪)-2021-0012), and experiments were approved by the Animal Ethics Committee of the Shanghai Tenth People's Hospital, Tongji University School of Medicine (Accreditation number of the laboratory: SHDSYY-2021-1729). The experiment was performed on 6-weeks old female ICR mice (n = 10) using the previously reported method.[12] $PDAP_{20}$ was prepared in sterile saline at a concentration of 10 mg/mL. AmpB was prepared in DMSO and further diluted into sterile saline to a final concentration of 0.3 mg/mL (2.5% DMSO in final solution). The injection doses of $PDAP_{20}$ and AmpB were 100 mg/kg and 3 mg/kg, respectively, and mice injected with saline were used as controls. After a single injection, all survival mice were weighed daily for 14 days. The organs (kidney, liver, and spleen) (n = 2) and blood of mice treated by $PDAP_{20}$ or saline (n = 8) were collected on the 14th day for histological analysis and blood biochemistry analysis.

Ocular toxicity. Male BALB/c mice (6-8 weeks old) were used for ocular toxicity studies (n = 3). The mice were randomly grouped and three mice were tested in each group. $PDAP_{20}$ at 15 mg/mL in saline was administered to the eyes every 5 min during the first hour and every 30 min during the next 7 h. All mice were killed 30 min after the administration of the last eye drop. The treated eyeballs were collected and fixed in 4% paraformaldehyde. The fixed eyeballs were embedded in paraffin, sectioned, and stained with hematoxylin and eosin (H&E). The eyeballs treated with saline were used as the control.

***C. albicans* biofilm formation on contact lenses.** The contact lenses (Biomedic®, Cooper Vision) were cut into discs with a diameter of 3.5 mm and socked into 150 µL YPD medium at 37 °C overnight in individual wells of a 96-well plate. Then the discs were transferred to a new well containing 150 µL *C. albicans* SC5314 suspension at 1.0×10^7 CFU/mL in YPD and incubated at rt with shaking at 100 rpm. After 6 hours of incubation, the suspension was aspirated and the lenses were washed with PBS to remove any non-adhered cells, followed by

addition of 150 μL fresh RPMI medium into each well. The plate containing the contact lenses with adhered fungal cells was incubated at rt under shaking at 100 rpm for 24 h to form biofilms, which was used to infect the cornea in the keratitis model.

In vivo anitfugal efficacy study. Keratitis model was established using previously reported method with modifications.[13] Briefly, male BALB/c mice (6-8 weeks old) were intraperitoneally injected with cyclophosphamide (one dose of 150 mg/kg at the beginning and another dose of 100 mg/kg on day 4) for immunosuppression. On day 5, all mice were anesthetized via intraperitoneal injection of sodium pentobarbital (75 mg/kg). Additional anesthesia of the mice eyes was performed using 0.5% lidocaine hydrochloride. A 2 mm-diameter filter paper disc contained 1 μL 99% 1-heptanol was placed on the center of the cornea for 15 min. The corneal epithelium was ulteriorly scrape off by an iris repository and the eyes were irrigated with 10 mL of normal saline to remove any remaining 1-heptanol. A 3.5 mm-diameter contact lens with *Candida albicans* biofilm was then placed on the cornea surface. Then the lid was closed with 8-0 sutures to make the contact lens inside fit on the surface of the cornea. After 18 h inoculation, the eyelid was opened and the lens was removed. The mice with keratitis were randomly divided into the following three treatment groups with each group consisting of 6 replicates: saline (the negative control), 15 mg/mL of $PDAP_{20}$ and 1 mg/mL of AmpB (the positive control). 10 μL eye drop was applied to each mouse every 5 min during the first hour and every 30 min during the next 7 h. All mice were killed and the eyeballs of each mouse were collected immediately 30 min after the administration of the last eye drop. Ten eyeballs from each group were homogenized for quantitative analysis of fungal counts with the number of CFUs expressed as mean log (CFU per eye), and the remaining four eyeballs were collected for histological analysis. The eyeball homogenate was serially diluted to for fungal cell culture and CFU counting on sabouraud dextrose agar (SDA) plates. After incubation for 24 h at 30 °C, the colonies were counted and the number of CFUs was

expressed as mean $\log_{10}$ (CFU per eye). The fixed eyeballs were embedded in paraffin, sectioned, and stained with periodic acid-schiff (PAS) for histological analysis, which stains fungal cells in purple. All images were scanned in bright field Pannoramic 250/MIDI equipped with the CaseViewer 2.4 software.

Statistical Analysis. Statistical analysis was performed with Origin software. Significance between two groups was determined by two-tailed t-test. One-way analysis of variance (ANOVA) with Tukey post-test for more than two variables was carried out. All results were expressed as mean values ± s.d. n = 3 for each in vitro statistical analysis. n = 8 or 10 for each in vivo statistical analysis.

Table S1. MIC values of $PDAP_{20}$ and ε-PL against *C. albicans* K1 under various salt concentrations.

Condition	Salt concentration (mM)		MIC (μg/mL)	
	Na^+	K^+	$PDAP_{20}$	ε-PL
Standard MIC test in RPMI 1640	103	5.4	0.8	400
Physiological salt concentration	150	5.4	1.6	1600
Na^+ ↑	200	5.4	3.1	>1600
Na^+ ↑	300	5.4	6.3	>1600
K^+ ↑	103	10	0.8	400
K^+ ↑	103	20	0.8	400
Na^+ ↑, K^+ ↑	300	20	6.3	>1600

Table S2. Antimicrobial activities of $PDAP_{20}$ and ε-PL.

Microorganisms		MIC (μg/mL)		
		$PDAP_{20}$	ε-PL	Positive control*
Gram positive	*S. aureus*	>200	3.1	0.4
	S. epidermidis	200	3.1	0.4
	B. subtilis	50	1.6	0.2
Gram negative	*E. coli*	200	6.3	0.2
	K. pneumoniae	>200	6.3	0.8
	A. baumannii	>200	6.3	0.2

* The positive controls used in Gram positive and Gram negative bacteria are vancomycin and polymyxin B, respectively.

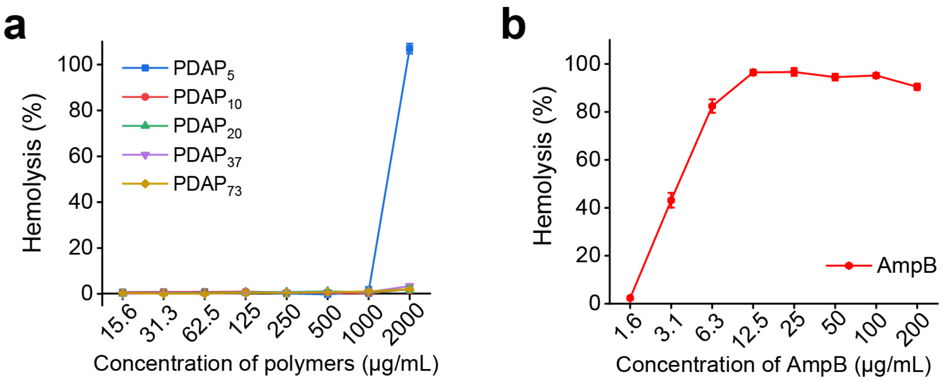

Figure S1. Hemolytic activity of PDAPs (**a**) and AmpB (**b**) on human red blood cells. n = 3, mean value ± s.d.

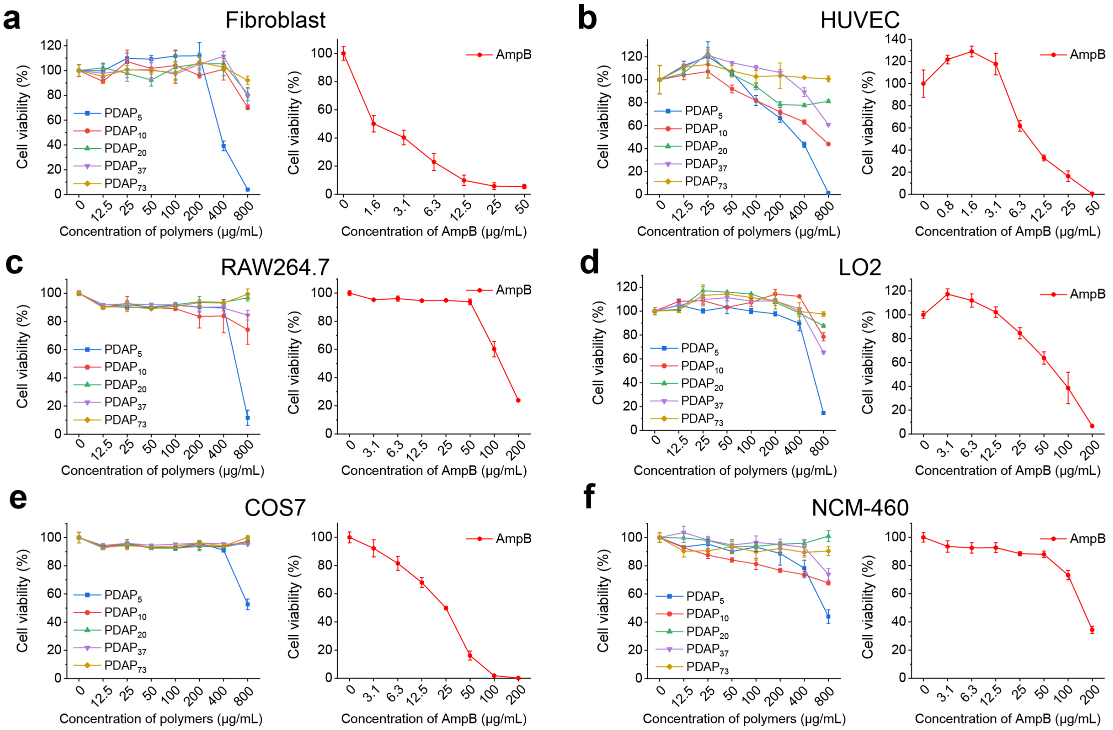

Figure S2. MTT-viability assay of NIH 3T3 fibroblasts (**a**), human umbilical vein endothelial cells (HUVEC) (**b**), macrophage RAW264.7 (**c**), liver cells (LO2) (**d**), kidney cells (COS7) (**e**), enterocyte (NCM-460) (**f**) in the presence of PDAPs and AmpB. n = 3, mean value ± s.d.

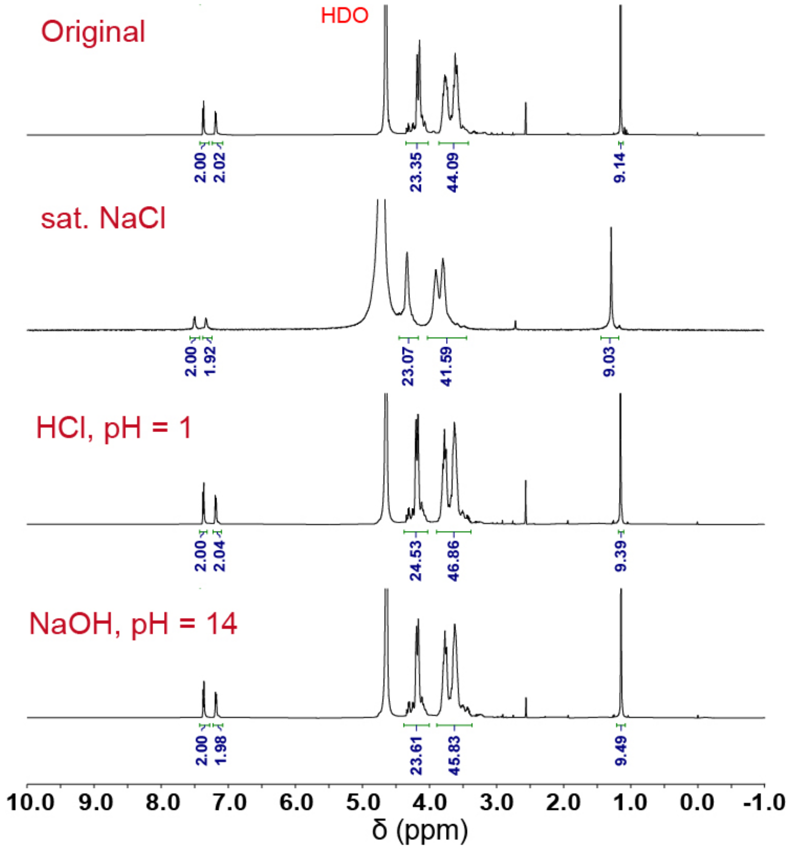

Figure S3. Stability test of $PDAP_{20}$ under saturated NaCl, acid (pH = 1) or basic (pH = 14) conditions as measured by ^1H NMR.

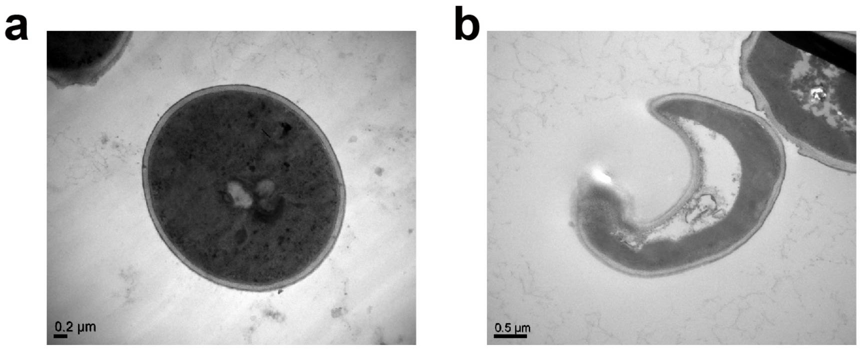

Figure S4. TEM micrograph of a cross-section of *C. albicans* K1 strain before (**a**) and after (**b**) $PDAP_{20}$ treatment.

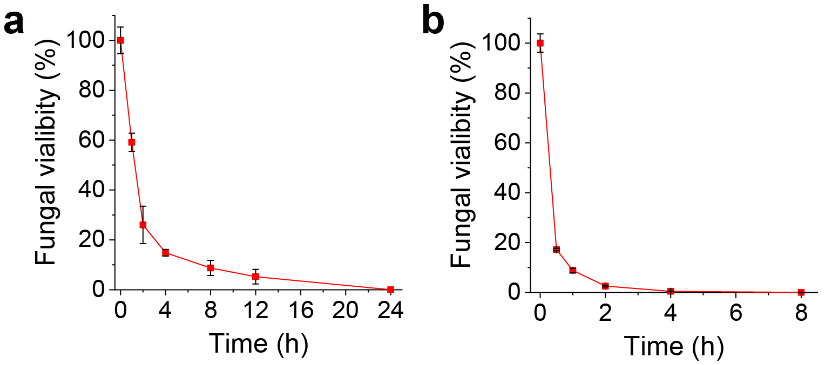

Figure S5. Time-kill kinetics of $PDAP_{20}$ at 2 × MFC against K1 strain under fungal concentration of 1250 CFU/mL (**a**) and 3.0 × 10^6 CFU/mL (**b**). n = 3, mean value ± s.d.

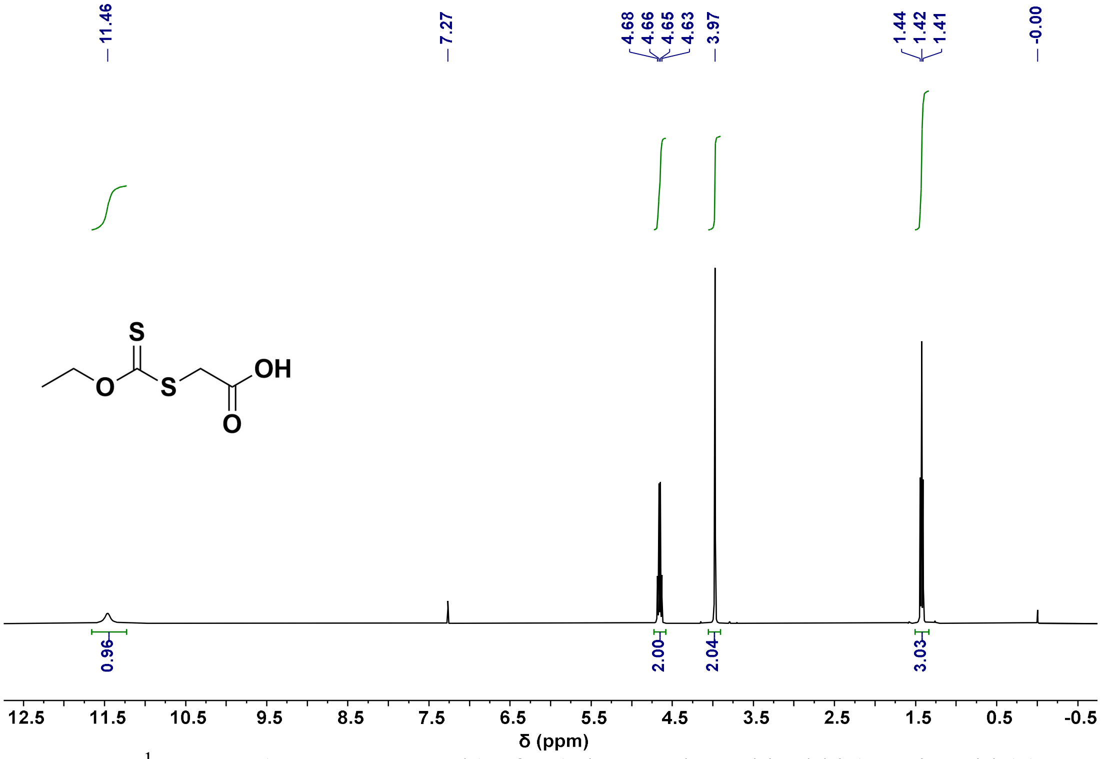

Figure S6. ^{1}H NMR (400 MHz, $CDCl_3$) of 2-(ethoxycarbonothioylthio)acetic acid (**1**).

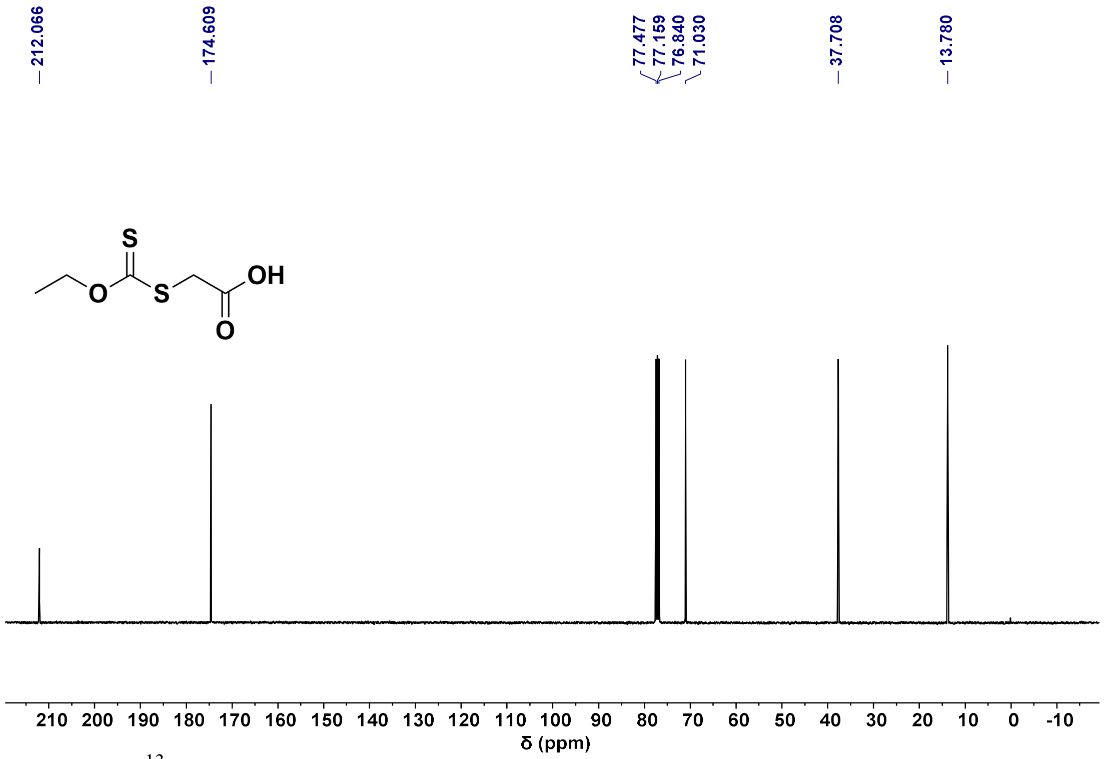

Figure S7. ^{13}C NMR (100 MHz, $CDCl_3$) of 2-(ethoxycarbonothioylthio)acetic acid (**1**).

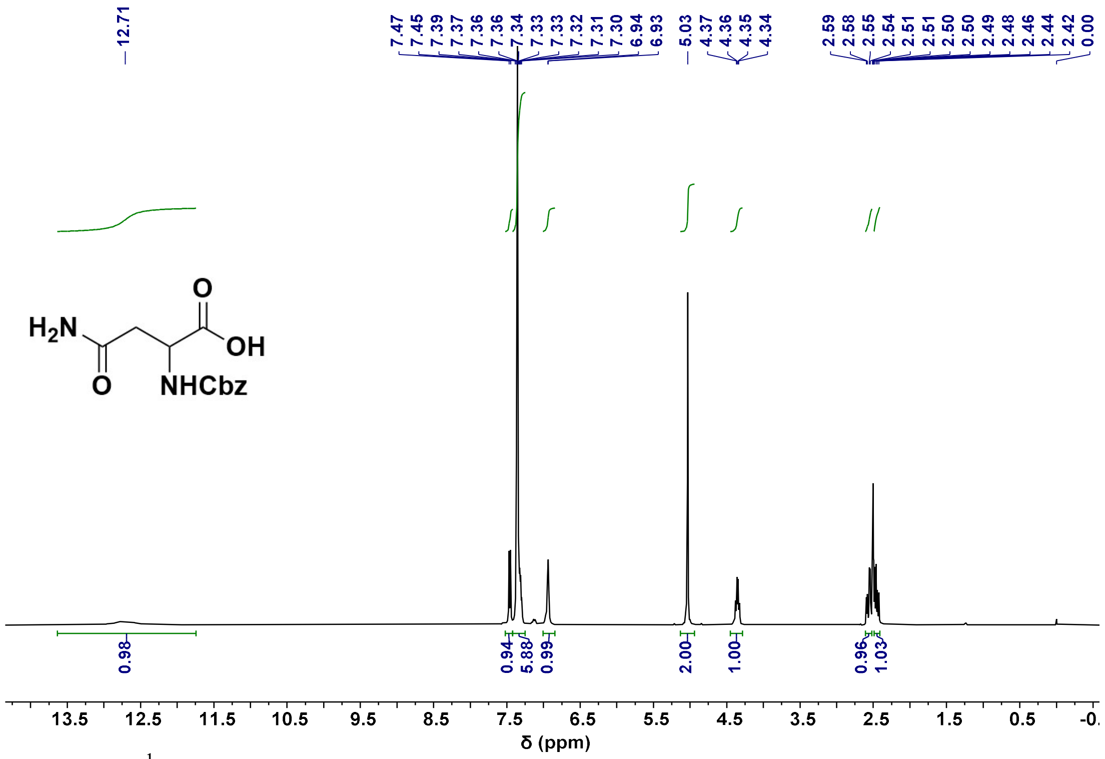

Figure S8. ^{1}H NMR (400 MHz, DMSO-d6) of N-Carbobenzoxy-DL-asparagine (**2**).

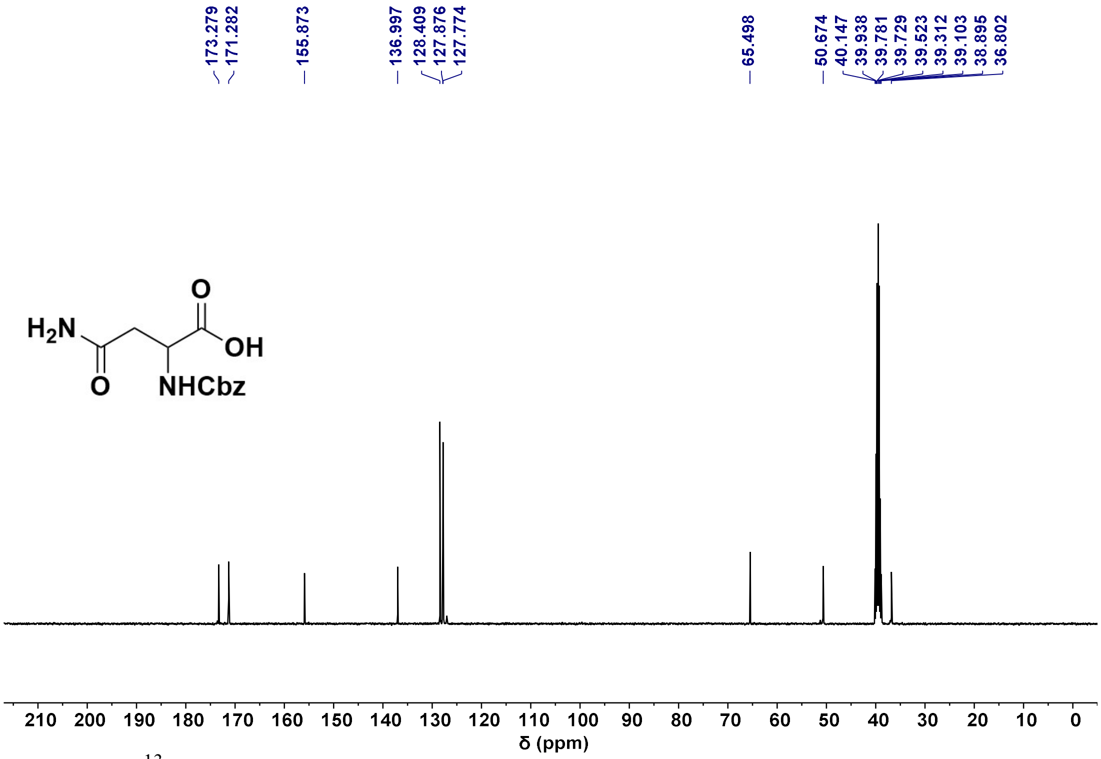

Figure S9. ^{13}C-NMR (100 MHz, DMSO-d6) of N-Carbobenzoxy-DL-asparagine (**2**).

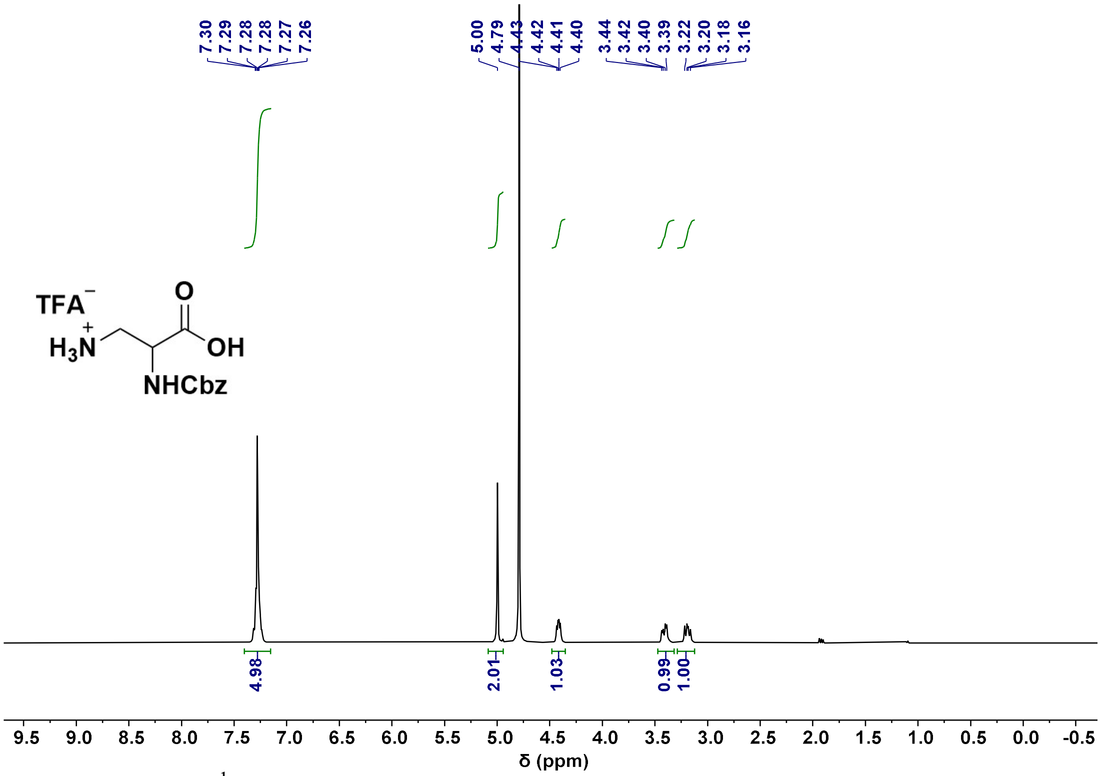

Figure S10. ^{1}H NMR (400 MHz, D_2O:TFA = 5:1) of (±)-3-Amino-2-benzyloxycarbonylaminopropanoic acid (**3**).

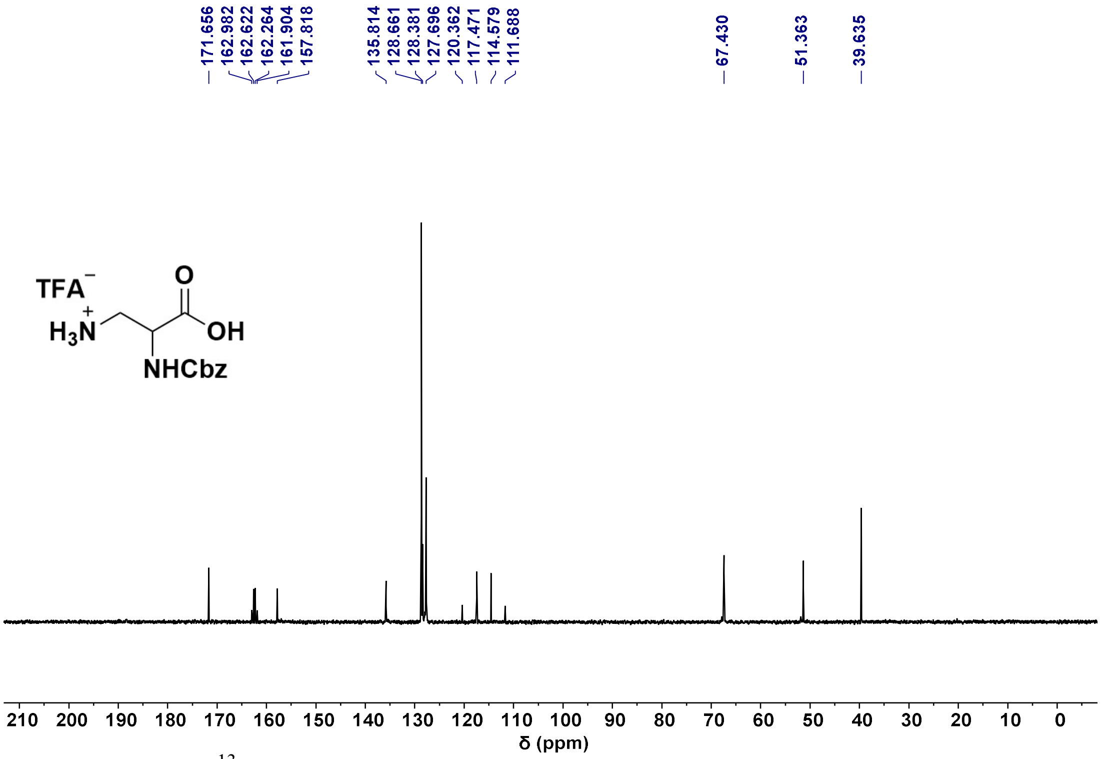

Figure S11. ^{13}C NMR (100 MHz, D_2O:TFA = 5:1) of (±)-3-Amino-2-benzyloxycarbonylaminopropanoic acid (**3**).

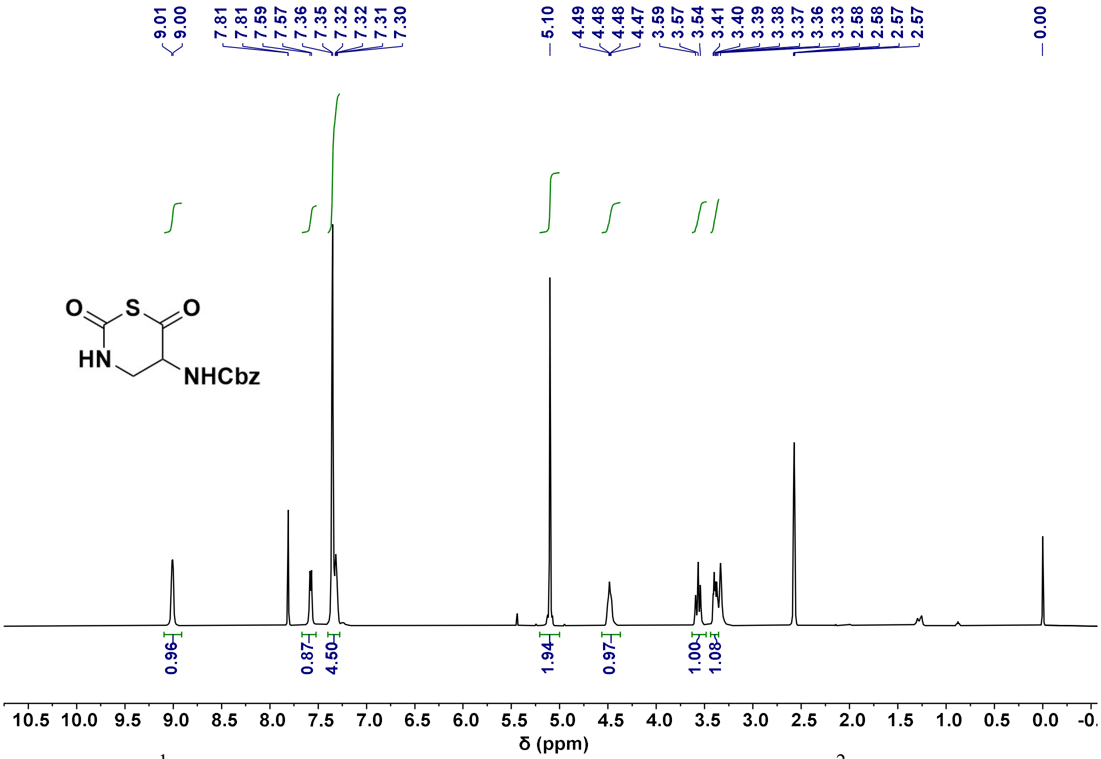

Figure S12. ^{1}H NMR (500 MHz, $CDCl_3$: DMSO-d6 = 2:1) of Cbz-β^2-DLDAP (**5**).

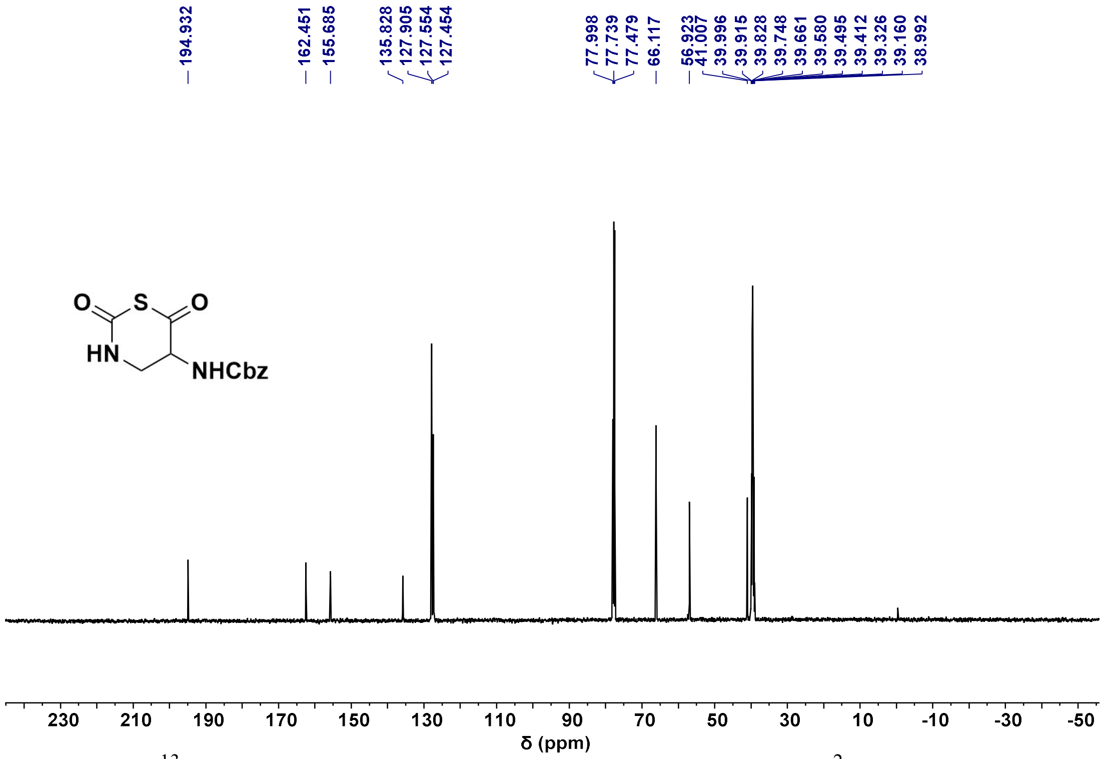

Figure S13. ^{13}C NMR (125 MHz, $CDCl_3$:DMSO-d6 = 2:1) of Cbz-β^2-DLDAP (**5**).

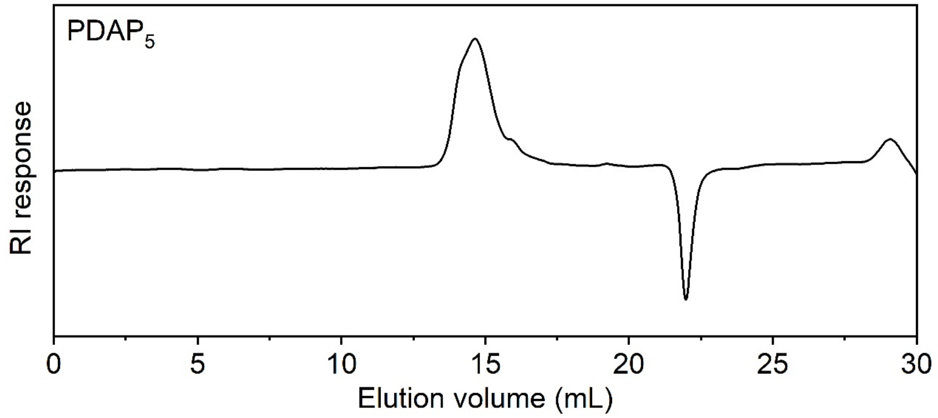

Figure S14. GPC trace of $PDAP_5$ at the sidechain protected stage using DMF as the mobile phase.

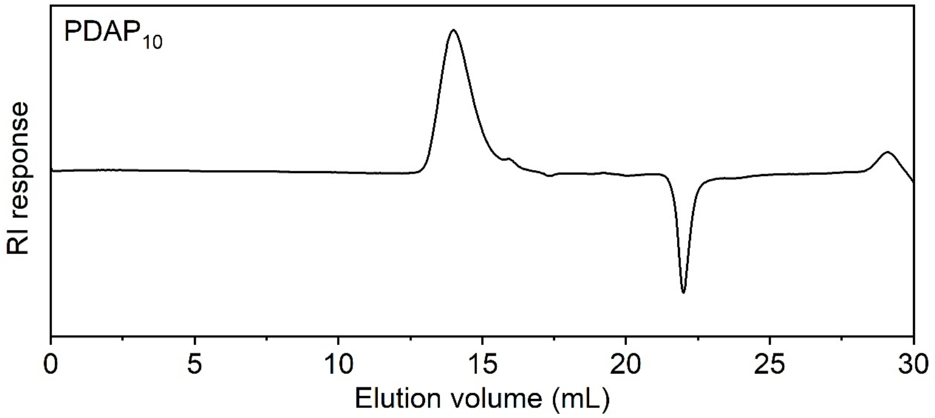

Figure S15. GPC trace of $PDAP_{10}$ at the sidechain protected stage using DMF as the mobile phase.

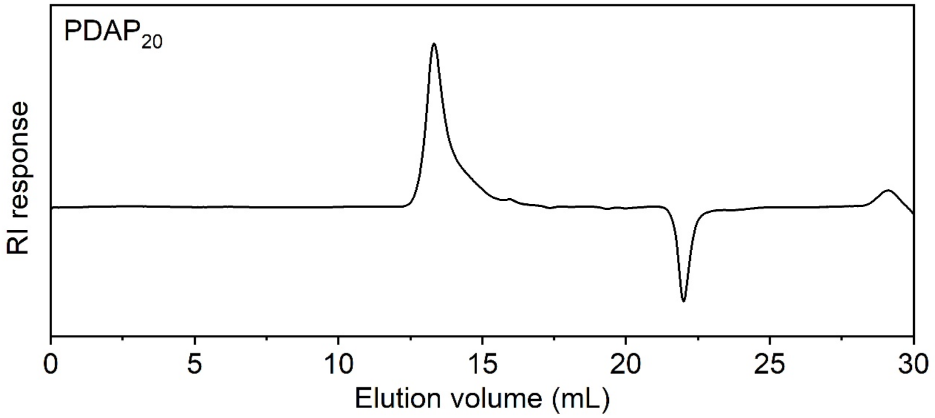

Figure S16. GPC trace of $PDAP_{20}$ at the sidechain protected stage using DMF as the mobile phase.

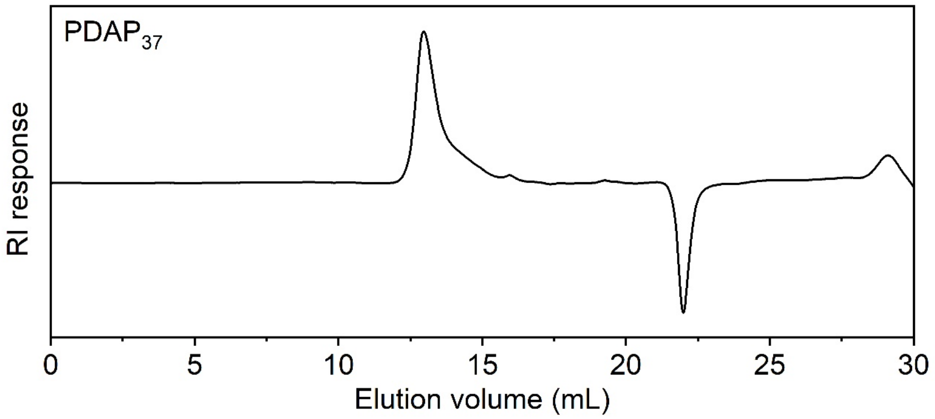

Figure S17. GPC trace of $PDAP_{37}$ at the sidechain protected stage using DMF as the mobile phase.

[1] M. Zhou, X. Xiao, Z. Cong, Y. Wu, W. Zhang, P. Ma, S. Chen, H. Zhang, D. Zhang, D. Zhang, X. Luan, Y. Mai, R. Liu, *Angew Chem Int Ed Engl* **2020**, *59*, 7240-7244.

[2] S. K. Ghosh, C. Ganzmann, J. A. Gladysz, *Tetrahedron: Asymmetry* **2015**, *26*, 1273-1280.

[3] R. Liu, X. Chen, Z. Hayouka, S. Chakraborty, S. P. Falk, B. Weisblum, K. S. Masters, S. H. Gellman, *J. Am. Chem. Soc.* **2013**, *135*, 5270-5273.

[4] R. Liu, X. Chen, S. P. Falk, K. S. Masters, B. Weisblum, S. H. Gellman, *J. Am. Chem. Soc.* **2015**, *137*, 2183-2186.

[5] a) V. Duncan, D. Smith, L. Simpson, E. Lovie, L. Katvars, L. Berge, J. Robertson, S. Smith, C. Munro, D. Mercer, D. O'Neil, *Antimicrob. Agents Chemother.* **2021**, *65*, e0234520; b) W. Chang, J. Liu, M. Zhang, H. Shi, S. Zheng, X. Jin, Y. Gao, S. Wang, A. Ji, H. Lou, *Nat. Commun.* **2018**, *9*, 5102; c) L. P. Menzel, H. M. Chowdhury, J. A. Masso-Silva, W. Ruddick, K. Falkovsky, R. Vorona, A. Malsbary, K. Cherabuddi, L. K. Ryan, K. M. DiFranco, D. C. Brice, M. J. Costanzo, D. Weaver, K. B. Freeman, R. W. Scott, G. Diamond, *Sci. Rep.* **2017**, *7*, 4353; d) S. Lin, W. L. W. Sin, J. J. Koh, F. Lim, L. Wang, D. Cao, R. W. Beuerman, L. Ren, S. Liu, *J. Med. Chem.* **2017**, *60*, 10135-10150.

[6] J. Han, M. A. Jyoti, H. Y. Song, W. S. Jang, *PLoS One* **2016**, *11*, e0150196.

[7] B. Zhao, D. Yang, J. H. Wong, J. Wang, C. Yin, Y. Zhu, S. Fan, T. B. Ng, J. Xia, Z. Li, *Chembiochem* **2016**, *17*, 1416-1420.

[8] A. Lupetti, A. Paulusma-Annema, S. Senesi, M. Campa, J. T. Van Dissel, P. H. Nibbering, *Antimicrob. Agents Chemother.* **2002**, *46*, 1634-1639.

[9] H. Tian, S. Qu, Y. Wang, Z. Lu, M. Zhang, Y. Gan, P. Zhang, J. Tian, *Appl. Microbiol. Biotechnol.* **2017**, *101*, 3335-3345.

[10] H. Choi, J. S. Hwang, D. G. Lee, *Insect. Mol. Biol.* **2014**, *23*, 788-799.

[11] C. G. Pierce, P. Uppuluri, A. R. Tristan, F. L. Wormley, Jr., E. Mowat, G. Ramage, J. L. Lopez-Ribot, *Nat. Protoc.* **2008**, *3*, 1494-1500.

[12] A. Halperin, Y. Shadkchan, E. Pisarevsky, A. M. Szpilman, H. Sandovsky, N. Osherov, I. Benhar, *J. Med. Chem.* **2016**, *59*, 1197-1206.

[13] K. Fukushima, S. Liu, H. Wu, A. C. Engler, D. J. Coady, H. Maune, J. Pitera, A. Nelson, N. Wiradharma, S. Venkataraman, Y. Huang, W. Fan, J. Y. Ying, Y. Y. Yang, J. L. Hedrick, *Nat. Commun.* **2013**, *4*, 2861.
